# Stepwise Hydride Transfer in a Biological System: Insights into the Reaction Mechanism of the Light‐Dependent Protochlorophyllide Oxidoreductase

**DOI:** 10.1002/anie.201712729

**Published:** 2018-02-05

**Authors:** Nataliya Archipowa, Roger J. Kutta, Derren J. Heyes, Nigel S. Scrutton

**Affiliations:** ^1^ Manchester Institute of Biotechnology and School of Chemistry The University of Manchester 131 Princess Street Manchester M1 7DN UK; ^2^ Current address: Institut für Physikalische und Theoretische Chemie Universität Regensburg Universitätsstr. 31 93053 Regensburg Germany

**Keywords:** photo-biophysics, photocatalysis, protochlorophyllide, stepwise hydride transfer, time-resolved spectroscopy

## Abstract

Hydride transfer plays a crucial role in a wide range of biological systems. However, its mode of action (concerted or stepwise) is still under debate. Light‐dependent NADPH: protochlorophyllide oxidoreductase (POR) catalyzes the stereospecific trans addition of a hydride anion and a proton across the C_17_−C_18_ double bond of protochlorophyllide. Time‐resolved absorption and emission spectroscopy were used to investigate the hydride transfer mechanism in POR. Apart from excited states of protochlorophyllide, three discrete intermediates were resolved, consistent with a stepwise mechanism that involves an initial electron transfer from NADPH. A subsequent proton‐coupled electron transfer followed by a proton transfer yield distinct different intermediates for wild type and the C226S variant, that is, initial hydride attaches to either C_17_ or C_18_, but ends in the same chlorophyllide stereoisomer. This work provides the first evidence of a stepwise hydride transfer in a biological system.

Hydride transfers (H^−^T) play a crucial role in a wide range of biological and chemical systems. Over 400 enzyme‐catalyzed H^−^T reactions depend on the cofactor nicotinamide adenine dinucleotide (phosphate), NAD(P)H, which acts as a source of two electrons and one proton (equivalent to a hydride ion).^1^ Therefore, reaction with a substrate results in the oxidized form, NAD(P)^+^, and the reduced substrate.^2^ In theory, H^−^T consists of three elementary steps as shown in Scheme 1.^1^, ^3^ Depending on the rate constants involving the necessary electron transfer (eT) and proton transfer (PT) reactions, the H^−^T can be entirely stepwise, partially stepwise, or purely concerted. The formation of radical pair intermediates can only be detected if the rate of the first eT is significantly different to the rate of the second eT, which creates a closed‐shell system again. However, owing to the elusive nature of short‐lived radical pair intermediates, direct evidence of the stepwise reaction mechanism is sparse. So far, direct detection of radicals during H^−^T has only been reported using NADH analogues as hydride donors and quinone derivatives or non‐heme oxoiron(IV) complexes as hydride acceptors in thermally driven reactions.^4^, ^5^, ^6^ Owing to the limitations imposed by the necessity to use rapid mixing strategies to initiate catalysis in thermally activated enzymes, no such mechanistic insights could be obtained yet.

**Scheme 1:**
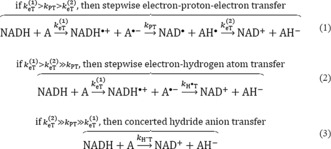
Elementary reaction steps involved in the H^−^ transfer reaction.

Light‐activated enzymes, where the reaction chemistry can be triggered by a short laser pulse, allow the study of H^−^T with a much better time resolution. The light‐dependent NADPH:protochlorophyllide oxidoreductase (POR) catalyzes the reduction of the C_17_−C_18_ double bond in ring D of protochlorophyllide (PChlide, P) to produce chlorophyllide (Chlide, C).^7^ The enzyme requires the coenzyme NADPH^8^ while the substrate PChlide acts as a photoreceptor (Figure 1).^9^ The double bond reduction was shown to proceed in a sequential manner^10^, ^11^ on a μs timescale, where first H^−^T from the *pro‐S* face of NADPH to the C_17_ position of PChlide^12^, ^13^ occurs followed by a PT to C_18_, most likely from a conserved Tyr residue within the POR active site.^14^ Recently, an alternative mechanism was proposed for the active site mutant POR‐C226S,^15^ in which after an initial eT a concerted hydrogen atom and PT transfer complete the double bond reduction.^15^ Although this suggests that stepwise H^−^T mechanisms may be possible, there is no direct evidence for this mechanism and the PChlide^.−^ and counter NADPH^.+^ radicals have yet to be identified.

**Figure 1:**
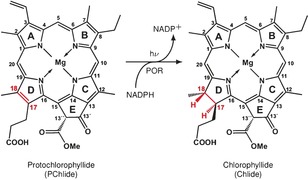
Light‐driven protochlorophyllide reduction catalyzed by the enzyme protochlorophyllide oxidoreductase, using NADPH as a coenzyme.

As PChlide acts as a photoreceptor, the POR‐catalyzed reaction also depends on the excited state dynamics of the substrate itself. Based on photophysical studies on (un)bound PChlide, different (branched^16^, ^17^, ^18^, ^19^, ^20^, ^21^ and sequential^22^, ^23^) models were proposed all starting from the excited singlet state, S_1_. Both models include the formation of an intramolecular charge transfer state (S_ICT_) that is suggested to induce an electron‐deficient site at the C_17_−C_18_ double bond of PChlide and facilitate its selective reduction. However, the proposed dynamics of the S_ICT_ differ and need further clarification (a detailed discussion on the individual models can be found in the Supporting Information). As link between the excited state dynamics of PChlide and POR chemistry, it was suggested that the S_ICT_ branches into a non‐catalytic and a catalytic route.^24^ In this model, while the former decays back into the ground state via formation of the solvated S_ICT_ and triplet, the latter leads to the formation of the reactive S_ICT_ followed by H^−^T and PT. However, whether the reactive S_ICT_ is directly linked to POR photochemistry or whether short‐lived intermediates, for example, radical pairs, precede the H^−^T reaction remains unclear.

We re‐investigated by transient absorption (TA) and emission the photophysics and photochemistry of PChlide unbound or bound to POR. We use the following nomenclature: ^*S*^P_*n*_=PChlide, ^*S*^I^*(x)*^_*n*_=intermediates arising from (photo)chemistry between excited PChlide and NADPH, and ^*S*^C_*n*_=Chlide, where *S* is the corresponding spin multiplicity, *n* the electronic state number, and *x* the number of an individual intermediate.

As a detailed understanding of the PChlide photophysics is essential for the interpretation of the photochemistry catalyzed by POR, we begin with a brief overview of the light‐induced dynamics obtained for the unbound PChlide and when bound in the non‐productive pseudoternary complex, PChlide/POR/NADP^+^. An extended discussion and corresponding data can be found in the Supporting Information. Excitation of PChlide, either unbound or bound to POR in PChlide/POR/NADP^+^ (ACN/Buffer/non‐productive complex), results in dynamics that follow standard photophysics with an excited singlet, ^1^P_1_, and a triplet state, ^3^P_1_, where the ^3^P_1_ is partially formed from the ^1^P_1_ and both states decay back into the ground state, ^1^P_0_ (green and orange frames in Figure 3 A). The ^1^P_1_ dynamics are accompanied by vibrational relaxation and solvent reorganization dynamics with typical lifetimes for those processes (16 ps/6 ps and 53 ps/4.2 ps). When excited into higher excited singlet states (for example, 450 nm), ^1^P_4_, the ^1^P_4_→^1^P_1_ transition is additionally observed with a fs lifetime (112 fs/142 fs/116 fs). The ^1^P_1_ decays with a ns lifetime (4.3 ns/4 ns/3.9 ns) into ^3^P_1_ and ^1^P_0_ with a triplet quantum yield of 50 %/55 %/55 %. The ^3^P_1_ decay shows an expected O_2_ dependency with total decay rates, *k*_T_, of (250 ns)^−1^/(4.8 μs)^−1^/(19 μs)^−1^ under aerobic and (170 μs)^−1^/(1 ms)^−1^/(73 μs)^−1^ under anaerobic conditions. Thus, the intrinsic back intersystem crossing rate, *k*_bisc_, given by the reciprocal lifetimes obtained from the data under anaerobic conditions, together with the concentration of O_2_ (*c=*2.4 mmol L^−1^ in ACN;^25^ *c=*0.25 mmol L^−1^ in H_2_O^26^) gives a O_2_‐quenching rate of *k*_q_^O2^=(*k*_T_−*k*_bisc_)/[O_2_]=1.7×10^9^ L mol^−1^ s^−1^/8.3×10^8^ L mol^−1^ s^−1^/10×10^8^ L mol^−1^ s^−1^. Interestingly, in the non‐productive complex, the O_2_‐quenching rate is five times smaller compared to *k*_q_^O2^ found for unbound PChlide in buffer. This demonstrates a protective role of POR against reactions with O_2_ within the lifetime of the ^3^P_1_, thus avoiding the formation of reactive oxygen species as long as the individual intermediates within the chlorophyll biosynthesis cannot be found unbound in vivo and are rather transferred from enzyme to enzyme. The characterization of the standard photophysics (green and orange frames in Figure 3 A) allows the determination of characteristic and distinct different species associated spectra (SAS) and their corresponding dynamics for ^1^P_1_ and ^3^P_1_ (Figure 3 B,C). These are the basis for the study on the productive ternary complexes with NADPH instead of NADP^+^.

We then investigated the productive ternary complex, PChlide/POR/NADPH (Supporting Information, Figure S10 A). In the first 50 ps after excitation, the relaxation dynamics are comparable to those observed in the non‐productive complex (Supporting Information, Figure S11 A, B1,2), indicating no significant fast change in the electronic state populations. However, on longer timescales the picture changes dramatically. On a sub‐ns timescale, the ^1^P_1_ decays faster compared to the non‐productive complex, and instead of the ^3^P_1_ spectrum, different TA features arise indicating the formation of a new electronic species with positive TA contributions at ca. 400, 477, and between 650 to 700 nm (Figure 2 A). Compared to the non‐productive complex, the ^1^P_1_ is quenched from 3.9 ns to 650 ps resulting in a quantum yield of 83 % (*Φ*(^2^I_0_^(1)^)=1−*τ*/*τ*_0_, where *τ*_0_ and *τ* are the ^1^P_1_ lifetimes of the (non‐)productive ternary complexes) for the first intermediate. On a sub‐100 ns timescale, a second intermediate with a more intense absorption at ca. 477 nm and an additional small but distinct absorption band at ca. 760 nm (Figure 2 C) is observed. To note, on this timescale we do not observe the formation of ^3^P_1_, and thus *k*_isc_ is negligible in this case. Further, a third intermediate is observed with a characteristic absorption band at ca. 700 nm, which finally decays into the product Chlide, ^1^C_0_, with its characteristic absorption band at ca. 680 nm (Figure 2 C), as reported.^10^ The TA data can be globally fitted with five exponentials yielding decay associated difference spectra (DADS) with well‐separated lifetimes, *τ_i_*, of 650 ps, 86 ns, 926 ns, 129 μs, and ∞ s (Figure 2 E). Since the reduction of the C_17_−C_18_ double bond requires two electrons and two protons, the observed lifetimes are consistent with individual single steps including two eTs and two PTs. PChlide, even in its excited singlet state, lacks the characteristics of a base so that we can exclude a PT as the initial reaction step (for a more detailed discussion, see the Supporting Information). Thus, we interpret the first intermediate as a radical anion (^2^I_0_^(1)^) after eT from NADPH to ^1^P_1_. Consequently, PT is the most probable step to follow, neutralizing the ionic radical pair. However, we observe two similar DADS with lifetimes separated by a factor of ca. 10, which are prolonged when the deuterated cofactor NADPD is used (Supporting Information, Figure S12 A,B), indicating that in the formation of the second and third intermediate a H/D atom transfer from NADPH/D is involved. Thus, steps 2 and 3 are consistent with a proton coupled eT forming ^2^I_0_^(2)^ and ^1^I_0_^(3)^, respectively. ^1^I_0_^(3)^ represents a single addition of hydrogen to the PChlide anion, which is finally protonated from either surrounding H_2_O molecules or acidic amino acid residues inside the protein pocket, yielding the final product ^1^C_0_. The recombination in the mechanistic sequence of the individual intermediates into their ground‐state species is always allowed. Figure 3 A (red frame) summarizes the proposed reaction mechanism starting from the excited PChlide singlet state. This model was applied onto the TA data resulting in reasonable SAS for each intermediate (Figure 3 D) with corresponding yields of *Φ*(^2^I_0_^(1)^)=0.83, *Φ*(^2^I_0_^(2)^)=0.5, *Φ*(^1^I_0_^(3)^)=1.0, and *Φ*(^1^C_0_)=1.0. Thus, the total quantum yield of ^1^C_0_ formation is *Φ* ^total^(^1^C_0_)=*Φ*(^2^I_0_^(1)^) *Φ*(^2^I_0_^(2)^) *Φ*(^1^I_0_^(3)^) *Φ*(^1^C_0_)=*Φ*(^2^I_0_^(1)^)=0.43, which is in good agreement with published data.^28^ However, we were not able to resolve the counter NADP(H) cation and neutral radicals, although they are expected to absorb in the same spectral regions (*λ*_max_=370 and 550 nm and *λ*_max_=400 and 500 nm, respectively). Compared to PChlide, they have ca. 10‐fold smaller extinction coefficients ranging between 0.5 and 5×10^3^ L mol^−1^ cm^−1^,^29^ and, thus, are masked by the corresponding PChlide intermediates. Similar species were also reported for light‐independent POR.^30^ However, a direct comparison cannot be made owing to different environmental effects, that is, Coulombic interactions to an additional Fe–S cluster (for a more detailed discussion, see the Supporting Information).

**Figure 2:**
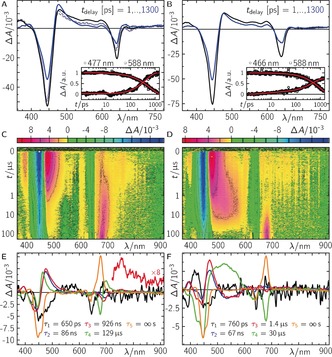
Photochemistry of the ternary complexes PChlide/NADPH/POR (A,C,E), and PChlide/NADPH/POR‐C226S (B,D,F) after excitation at *λ*_exc_=640 nm. A),B) Time‐resolved difference absorption spectra up to 1.3 ns. Dashed lines represent the corresponding data of the pseudo‐ternary complex for comparison. Insets in (A) and (B): selected time traces plus fit. C),D) False‐color plots of raw data up to 200 μs. The data in the gray dashed rectangles contained invalid data owing to laser scatter and were replaced by the fit during the global fitting.^27^ E,F) Decay associated difference spectra from global fits on data in (C) and (D).

**Figure 3:**
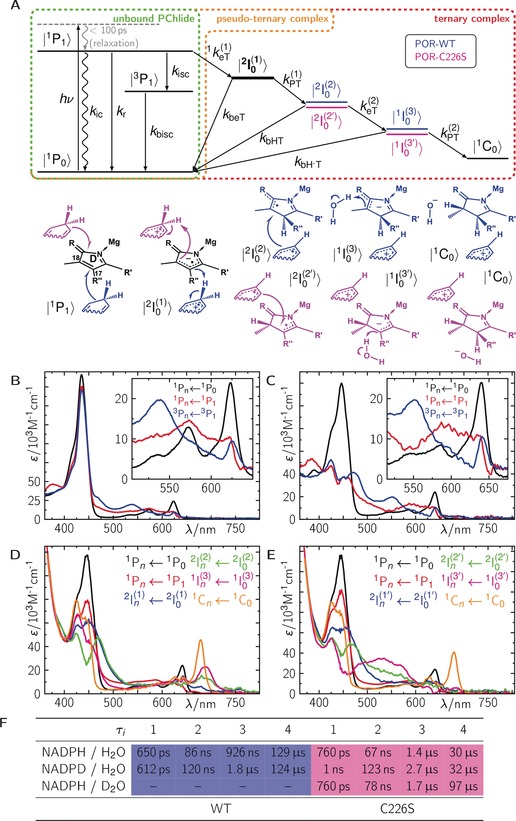
A) Photophysical/chemical model and B)–E) the corresponding species‐associated spectra for B) unbound PChlide, C) bound PChlide in the pseudoternary PChlide/NADP^+^/POR, D) the ternary PChlide/NADPH/POR, and E) PChlide/NADPH/POR‐C226S complexes. F) Lifetimes from global fits on data with wild type and the C226S mutant in the presence of either NADPH/H_2_O, NADPD/H_2_O, or NADPH/D_2_O. Abbreviations: *k*_r_=radiative decay; *k*_ic_=internal conversion; *k*_isc_=intersystem crossing; *k*_bisc_=back intersystem crossing; ^1^ *k*_eT_^(1)^=1st electron transfer; *k*_beT_=back electron transfer; *k*_PT_^(1)^=1st proton transfer; *k*_bHT_=back H atom transfer; *k*_eT_^(2)^=2nd electron transfer; *k* ${}_{\mathrm{bH}{}^{-}T}$ =back hydride transfer; *k*_PT_^(2)^=2nd proton transfer.

Finally, we investigated the ternary complex, PChlide/POR‐C226S/NADPH, which was previously proposed to proceed via an alternative reaction mechanism compared to wild type (WT).^15^ Here, ^1^P_1_ is quenched from 4.4 ns to 760 ps, resulting in a quantum yield of 83 % (see argument for WT data above) for the first eT forming ^2^I_0_^(1)^ which is identical to the situation in WT. Again, no triplet is observed following the ^1^P_1_ decay, indicating a negligible *k*_isc_. The following kinetics are slightly altered compared to WT (Figure 2 B,D,F). Especially, the final ^1^C_0_ formation is ca. 4‐fold faster in the mutant. Because 1) steps 2 and 3 become slower in the presence of NADPD; and 2) the last step becomes slower in the presence of D_2_O, the data for the mutant suggest similar chemical intermediates to those for WT. However, inspection of the involved spectral changes (Figure 2) reveals distinct differences for the second and third intermediate. Correspondingly, application of the same model as for WT (red frame in Figure 3 A) resolves distinct different SAS for the 2nd (^2^I_0_^(2′)^) and 3rd (^1^I_0_^(3′)^) intermediate, while the other SAS agree well within experimental error to the ones obtained for WT (Figure 3 E). Here, the total quantum yield of ^1^C_0_ formation is *Φ* ^total^(^1^C_0_)=0.44. Heavy atom experiments showed that identical chemical reaction partners are involved. Thus, we conclude that the formed PChlide species in POR‐C226S must differ compared to those formed in WT, for example, by attachment of the hydride at C_18_ rather than C_17_. Quantum‐chemical calculations of all possible stereoisomers on a qualitative basis indeed show that the absorption spectrum of H‐C_18_ (=^1^I_0_^(3′)^) is expected to have a distinct broad absorption band at ca. 550 nm, which is more intense than for H‐C_17_ (=^1^I_0_^(3)^). Further, the expected band at ca. 700 nm for ^1^I_0_^(3)^ resolves pretty well but is less intense and red‐shifted for ^1^I_0_^(3′)^ (Supporting Information, Figure S17). Finally, circular dichroism spectra of ^1^C_0_ either formed by WT or by POR‐C226S are identical, indicating the same stereoisomer for ^1^C_0_ (Supporting Information, Figure S18 D). Therefore, we conclude that PChlide is bound turned by 180° in the mutant compared to WT, allowing the formation of identical products (Figure 3 A; Supporting Information, Figure S18 A–C).

Here, we have investigated the mechanism of the light‐dependent POR, which reduces PChlide to Chlide. Apart from the photophysical states of (un)bound PChlide, that is, excited singlet and triplet, we were able to resolve three intermediates consistent with a stepwise hydride transfer mechanism, that is, interpreted as an initial eT from NADPH to the excited PChlide singlet state followed by a proton coupled eT and a subsequent PT. Identical transfer reactions were observed for the C226S variant, but with distinct different PChlide species in terms of hydride attachment at C_18_ instead of C_17_, which is potentially due to an altered PChlide binding. To our knowledge, this is the first biological system that allowed the direct observation of a stepwise hydride transfer. Our study provides general understanding of how light energy can be harnessed to drive H‐transfer chemistry, with implications for the design of light‐activated chemical and biochemical catalysts. Additionally, the study also emphasizes how mutagenesis can fundamentally alter the reaction path of enzyme catalyzed H‐transfer. Thus, our work provides important and new insight into fundamental mechanisms of H‐transfer in a biological system and challenges the inference that biological hydride transfers proceed exclusively through concerted mechanisms. However, for thermally activated systems the H^−^‐transfer mechanism remains elusive owing to the lack of time resolution.

## Conflict of interest

The authors declare no conflict of interest.

## Supporting information

As a service to our authors and readers, this journal provides supporting information supplied by the authors. Such materials are peer reviewed and may be re‐organized for online delivery, but are not copy‐edited or typeset. Technical support issues arising from supporting information (other than missing files) should be addressed to the authors.

Supplementary
