# Supplementary material for: Stepwise Hydride Transfer in a Biological System: Insights into the Reaction Mechanism of the Light‐Dependent Protochlorophyllide Oxidoreductase
Source: Angew Chem Int Ed Engl. 2018 Feb 5;57(10):2682–6. doi: 10.1002/anie.201712729 (PMC5861667; doi:10.1002/anie.201712729)
Supplement: Supplementary file 1 — Supplementary [file ANIE-57-2682-s001.pdf]

Angewandte Chemie
Eine Zeitschrift der Gesellschaft Deutscher Chemiker

Supporting Information

Stepwise Hydride Transfer in a Biological System: Insights into the Reaction Mechanism of the Light-Dependent Protochlorophyllide Oxidoreductase

Nataliya Archipowa[+], Roger J. Kutta[+], Derren J. Heyes, and Nigel S. Scrutton**

anie_201712729_sm_miscellaneous_information.pdf

Table of Contents

Table of Contents 2

Experimental Procedures 3

Results and Discussion 5

References 30

Author Contributions 31

Experimental Procedures

Sample preparation.
Acetonitrile (Sigma Aldrich), β-Nicotinamide adenine dinucleotide 2'-phosphate reduced tetrasodium salt (NADPH-Na_4, Melford Laboratories Ltd.), or β-Nicotinamide adenine dinucleotide phosphate disodium salt ($NADP^+$, Sigma-Aldrich) were used as purchased. PChlide was purified as described in[1]. Deuterated *pro*-S NADPH ((S)-4-^{2}H-NADPH) was purified and analyzed for chemical and isotopic purity as described[2]. The concentrations of PChlide, $NADP^+$ and NADPH or (S)-4-^{2}H-NADPH were calculated by the Lambert-Beer law using the molar extinction coefficients ε(630 nm) = 23955 $M^{-1}cm^{-1}$,[3] ε(260 nm) = 17800 $M^{-1}cm^{-1}$, and ε(340 nm) = 6200 $M^{-1}cm^{-1}$,[4] respectively.

cDNA encoding the POR protein from *Thermosynechococcus elongatus* BP-1 was subcloned into the expression vector pRSET-A (Invitrogen) *via BamHI* and *HindIII* restriction sites. The sequence of the gene was confirmed by DNA sequencing (MWG Eurofins). The protein was recombinantly expressed in *Escherichia coli* SoluBL21 (Amsbio) as described in.[5] The cells were disrupted by sonication on ice for 20 min, at 45% amplitude with 20 s pulses/min. The 6xHis-tagged protein was purified by Ni-IDA (Generon) affinity chromatography in Tris buffer (50 mM, pH = 7.5) containing 500 mM NaCl, 20 mM imidazole, 20 Vol% glycerol, and 0.1 Vol% Triton-X100. Non-specifically bound proteins were washed away with binding buffer containing 50 mM imidazole followed by elution of the column-bound protein with binding buffer containing 300 mM imidazole. The protein was then concentrated in an Amicon with a 10 kDa cut-off filter to the desired volume followed by buffer exchange by dialysis into the buffer used in the measurements (50 mM Tris, 300 mM NaCl, 40 Vol% glycerol, 0.1 Vol% Triton-X100, pH = 7.5). The homogeneity and purity of the isolated POR protein was verified by SDS-PAGE after each preparation using 12% polyacrylamide gels (Mini-Protean® TGX Stain-FreeTM Gel, BioRad). The purified protein was shock frozen in liquid nitrogen and stored at -80 °C until use.

Site-directed mutagenesis was performed as described in[6]. The recombinant POR-C226S mutant protein was expressed and purified as described for the wild type, except that the buffer used in the measurement contained 500 mM NaCl and was prepared with either H_2O or D_2O. Note, that unlike other publications on POR no reducing agents, such as 2-mercaptoethanol or DTT, were used.

The concentration of the proteins was determined by measuring the absorption at 280 nm. The calculated molar extinction of POR is ε(280 nm) = 42650 $M^{-1}cm^{-1}$ (based on the Protparam tool on the Expasy web server).

Anaerobic samples were prepared in a glove box (Belle Technology, UK Ltd). While unbound PChlide samples were dissolved in oxygen free solvents or buffers that were incubated in the glove box overnight, samples containing POR enzyme were applied to a gravity flow CentriPure desalting column (Generon) using an already oxygen free buffer as mobile phase.

Generally for each aerobic measurement sample volumes of 5 mL 16 μM PChlide, 50 mL of corresponding pseudo-ternary POR(200 μM)/PChlide(16 μM)/$NADP^+$(2 mM), and 300 mL ternary POR(200 μM)/PChlide(16 μM)/NADPH((S)-4-^{2}H NADPH, 200 μM) complexes were used. 600 μL of PChlide or pseudo-ternary complex (same composition as in the aerobic samples) were measured under anaerobic conditions since the experimental set-up does not allow for anaerobic flow of the sample. The integrity of the samples was checked before and after each measurement by stationary UV/Vis absorption spectroscopy (Cary 50, Agilent Technologies). All measurements were performed at room temperature (*ca.* 20 °C).

Stationary circular dichroism absorption spectroscopy.

5 mL samples containing PChlide, NADPH and either wildtype POR or the C226S variant dissolved in phosphate buffer (50 mM, pH = 7.5, 300 mM NaCl, 0.01Vol% Triton-X 100) were illuminated until no observable changes in the Chlide concentration occurred. Subsequently, the corresponding Chlide was extracted into di-ethylether, adjusted to OD ≈ 0.9 over 10 mm pathlength in the maximum of the Soret band and incubated at least 15 min at 4°C prior to the measurement without further purification. Unbound PChlide transferred into di-ethylether was measured as reference. Stationary circular dichroism absorption spectra were recorded using a Chirascan CD-Spectrophotometer (Applied Photophysics Ltd) at 4°C in the visible range from 400 to 800 nm with a pathlength of 10 mm. The di-ethylether background was subtracted from each spectrum. Chlide spectra were corrected for unreacted PChlide by subtracting the adjusted PChlide CD spectrum to the Chlide spectrum. Figure S18 **D** shows an average of 20 scans, respectively.

Sub-ps and Sub-ns Pump/Supercontinuum-Probe Spectroscopy.
A Ti-sapphire amplifier system (Spectra Physics Solstice Ace) was used to generate 800 nm with 6 mJ pulses at 1 kHz. One third of the 800 nm pulses were used to pump a collinear Optical Parametric Amplifier (OPA, TOPAS-PRIME, Light Conversion) with an associated NirUVis unit tuned to pump pulses centered at either *ca.* 450 nm (*ca.* 10 nm full width at half-maximum (FWHM), *ca.* 100 fs, *ca.* 150 nJ) or at *ca.* 640 nm (*ca.* 20 nm FWHM, *ca.* 100 fs, *ca.* 150 nJ). Exact excitation wavelengths are given in the captions of the figures. The supercontinuum white light probe pulses were generated by focusing a small fraction of the 800 nm pulses into a moving CaF_2 disc of 3 mm thickness giving a probe spectrum ranging from 340 to 820 nm. For the sub-ps time range a Helios spectrometer (Ultrafast Systems) was used equipped with a delay line up to 3 ns. For the sub-ns time range an Eos spectrometer (Ultrafast Systems) was used in which the supercontinuum probe pulses are generated by a fiber laser (LEUKOS) at 2 kHz with a spectrum ranging from 350 to 790 nm and are electronically delayed relative to the pump pulses covering time ranges up to 400 μs. Both spectrometers use the same two complementary fiber coupled high-speed spectrometers equipped with metal-oxide-semiconductor (CMOS) detectors for signal and reference recording. The pump and probe pulses were focused colinearly into the sample to spot sizes of *ca.* 200 μm and *ca.*100 μm FWHM, respectively. The relative polarizations between the pump and probe were set to the magic angle (54.71°) by a half-wave plate in the pump-beam path. The *ca.* 1.5 ps chirp of the white light was corrected for prior to data analysis using the coherent artefact as an indicator for time zero at each wavelength. Throughout the probe range, the spectral resolution was better than 4 nm and the temporal resolution was better than 100 fs or 500 ps in the sub-ps or sub-ns experiment, respectively. In the sub-ps experiment 10 individual scans with averaging 100 spectra per time point were typically recorded. The time axis - within total 500 points - was linear between -1 and 1 ps and logarithmic from 1 ps to the maximum time delay so that the same number of delay points from −1 to 1 ps as between 1 and 10 ps, 10 and 100 ps, *etc.* is produced ensuring that the dynamics on every time scale will have equal weighting in the fitting analysis. In the sub-ns experiment, similar logarithmically spaced time delays (in total 500) are set. Samples were cycled through a flow cell with a path length of 2 mm for pump and probe

beams (dimensions: 1 mm x 2 mm x 30 mm, Starna) by a peristaltic pump (400U/D1, Watson Marlow). 2-4 independent measurements with 90-120 min duration were performed and analyzed independently. The total turnover (PChlide→Chlide) in the case of productive ternary complexes was <10% under the used experimental conditions. The shown data correspond to one representative measurement. No smoothing or filtering procedures were applied to the data.

Sub-ns time resolved photon counting.
A Ti:Sapphire amplifier laser system (Libra, Coherent) provides pulsed laser light at 800 nm that feeds into an collinear OPA (TOPAS-C, Coherent) generating the excitation pulses at 450 nm with a pulse duration of typically 80 fs giving an instrument response function of *ca.* 20 ps on a 10 ns time window due to the electronic jitter. The fluorescence was collected at *ca.* 30° to the excitation path and detected with a combination of a spectrograph (200is, Bruker) and streak camera (C7700, Hamamatsu). The streak images were recorded on a CCD camera (ORCA-CR, Hamamatsu).

Description of vibrational and solvation relaxation.
Population changes between different relaxed electronic states is always accompanied by vibrational and solvation relaxation dynamics. On time scales, much longer than the vibrational and solvation relaxation dynamics, these dynamics can be neglected and the population changes can be described by individual species associated spectra with their corresponding concentration-time profile (or mathematically: spectra and concentration-time profiles are orthogonal to each other). However, on time scales, typically for vibrational and solvation relaxation dynamics, the orthogonal description does not hold anymore, since the vibrational and solvation relaxation manifests in slight and continuous changes over time in the absorption spectrum after photo-excitation. In order to account for these dynamics, which cannot be accounted for by a global fit with the orthogonality approximation, spectral ranges of interest, *e.g.* those that show best the spectral evolution, were parameterized by a linear combination of Gaussians. In other words, at each delay time, *t*, a sum of Gaussians as defined in Eq. 1 was fitted to a certain spectral region.

$$\Delta A(t,\lambda) = \sum_i \mathrm{G}_i(t,\lambda) = \sum_i \frac{a_i(t)}{\sigma_i(t)\sqrt{\frac{\pi}{2}}} \exp\left(-2\left(\frac{\lambda-\lambda_{0,i}(t)}{\sigma_i(t)}\right)^2\right) \quad (1)$$

In this way, spectral changes such as the width, $\sigma(t)$, the location of maxima, $\lambda_0(t)$, as well as the area, $a(t)$, of bands can be determined as a function of time.

Transient absorption data analysis and modelling.
SVD-based rank analysis and global fitting were performed using an in-house written program described previously.[7,8] Briefly, transient absorption data consist of a series of difference spectra recorded at a number of delay times, $\Delta A(t,\lambda)$. This can be represented as a rectangular matrix $\mathbf{\Delta A}$ of dimension $N_\mathrm{T}\times N_\mathrm{L}$ with matrix elements, $\Delta A_{ij} = \Delta A(t_i,\lambda_j)$, where each column of the matrix is a time trace for a fixed wavelength of N_T points, and each row is a spectrum at a given delay time of N_L points. Decomposition of this data matrix into a sum of products of one-dimensional functions can be achieved by a global fit. In the global fit, the assumption is made that the data can be modelled by a linear combination of products between spectra, $S_k(\lambda)$, and concentration-time profiles, $c_k(t)$, as seen in Eq. 2.

$$\Delta A_{ij} = \sum_{k=1}^{N} c_k(t_i) S_k(\lambda_j) = \sum_{k=1}^{N} c_{ik} S_{jk} \quad (2)$$

Eq. 2 can be approximated by matrix $\mathbf{D}$ in which the time profiles are represented as a linear combination of known analytic functions, $f_k(t)$:

$$D_{ij} = \sum_{k=1}^{N_\mathrm{C}} c_k(t_i) S_k(\lambda_j) \quad (3)$$

$$c_k(t) = \sum_{k=1}^{N_\mathrm{F}} f_l(t) X_{lk} \quad (4)$$

Here N_C is the number of distinct spectral species and N_F is the number of analytic time functions. By defining $\mathbf{C}$ as a $N_\mathrm{T}\times N_\mathrm{C}$ matrix with elements, $C_{ik} = c_k(t_i)$, and $\mathbf{F}$ as a $N_\mathrm{T}\times N_\mathrm{C}$ matrix with elements, $F_{il} = f_l(t_i)$, the model data matrix $\mathbf{D}$ can be written as:

$$\mathbf{D} = \mathbf{CS} = \mathbf{FXS} = \mathbf{FB} \quad (5)$$

In this definition, the k-th row of the matrix $\mathbf{B}$, with elements $B_{kj} = b_k(\lambda_j)$, corresponds to the spectral changes associated with the time function, $f_k(t)$. Using exponential decays (convoluted with the instrument response) as analytical function the corresponding spectra are called decay associated difference spectra (DADS). For given matrices $\mathbf{\Delta A}$ and $\mathbf{F}$ the linear least squares problem in Eq. 6

$$\chi^2 = \|\mathbf{\Delta A} - \mathbf{FB}\|^2 = \mathrm{Min} \quad (6)$$

can be solved efficiently by existing algorithms. Further optimization of χ^2 is achieved by optimizing the rate constants in $\mathbf{F}$ by a nonlinear least squares algorithm. The DADS and the corresponding rate constants are the unique result of this global fit and this treatment does not require any model for the kinetics involved in the transient processes. The number of exponentials to use in the global fit is determined by SVD-based rank analysis, which is described elsewhere.[9] By this approach the details of a model will be entirely defined in the matrix $\mathbf{X}$ that relates the actual species kinetics to the elementary function, $f_k(t)$. Depending on the model, the appropriate matrix, $\mathbf{X}$, can be chosen and the species associated spectra (SAS) in matrix $\mathbf{S}$ are calculated according to Eq. 7.

$$\mathbf{S} = \mathbf{X}^{-1}\mathbf{B} \tag{7}$$

This step does not change the χ^2 value found in the global fit and, thus, this procedure has the advantage that all interpretation is performed with the same quality of fit. An exemplary residual plot for a global exponential fit on data obtained for the ternary complex PChlide/-NADPH/POR is shown in Figure S21. The residuals obtained for all other data sets are of comparable quality.

***Molecular dynamic simulations and quantum chemical calculations*.**
An initial sequence alignment was made using the SWISS-MODEL workspace resulting in 50 template sequences.[10] Of these 50 templates the putative uncharacterized protein from *Mycobacterium paratuberculosis* (PDB code: 3RD5)[11] has the highest sequence identity (29.78) and the β-ketoacyl reductase (RhlG/NADP active-site complex) from *Pseudomonas aeruginosa* (PDB code: 2B4Q)[12] showed the best QMEAN (-3.69) value. Then, the program MODELLER 9.11 was used to generate a more refined homology model structure of full length POR from *Thermosynechococcus elongatus*, TsPOR.[13] The position of NADPH was taken as found in the β-ketoacyl reductase. The PChlide molecule was parameterized based on the gromos53a6 force field, manually docked based on intuitive chemical interactions found in the POR protein pocket and then energetically minimized using the Gromacs package.[14] Molecular dynamics simulations were performed on the ternary complex POR/NADPH/PChlide with a solvation box of minimum 1 nm around the protein and periodic boundary conditions. After energy minimization, the system was initially thermalized to 300 K for 100 ps using *NVT* dynamics, and the pressure was then equilibrated for 100 ps using *NPT* dynamics. The protein, NADPH, and PChlide were constrained during these steps. All constraints and pressure couplings were then switched off and the system relaxed using *NPT* at 250, 280, 290, and 300 K for 1 ns each. Finally, 100 ns of *NPT* dynamics were run at 300 K. Representative structures were chosen as those with the smallest root mean squared deviations for the PChlide heavy atoms relative to the average (Figure S 18 **A**-**C**).

Quantum-chemical calculations were performed with time-dependent density functional theory (TD-DFT) using the Firefly QC package[15], which is partially based on the GAMESS (US)[16] source code. The ground state geometries of four possible stereoconformers of the PChlide anion (${}^1\mathrm{I}_0^{(3)}$, ${}^1\mathrm{I}_0^{(3')}$, ${}^1\mathrm{I}_0^{(3'')}$and ${}^1\mathrm{I}_0^{(3''')}$ in Figure S17) were optimized with density functional theory (DFT). The B3LYP functional and the 6-31G(d) atomic basis set were used for all singlet state calculation. Since the B3LYP functional is not implemented for excited triplet states in the Firefly QM package, the BVWN5 functional was used instead. Solvent effects were taken into account with the polarized continuum model (PCM, Figure S19-20).

Results and Discussion

General kinetic model.
We used the analytic fitting function shown in Eq. 8 in the global lifetime analysis in order to determine the dynamics of each system

$$f_k(\mathrm{t}) = \sum_{i=0}^{2} \frac{\mathrm{d}^i}{\mathrm{d}t^i} g_{\mathrm{art}}(t-t_0) + \left(\delta(t) + \sum_{j=1}^{N} \exp\left(-\kappa_k t\right)\right) \otimes g_{\mathrm{app}}(t-t_0) \tag{8}$$

where $\otimes\, g_{\mathrm{app}}(t-t_0)$ indicates convolution with the apparatus function approximated by a Gaussian, $\delta(t)$ is the Dirac delta function, $\sum_{i=0}^{2}\frac{\mathrm{d}^i}{\mathrm{d}t^i} g_{\mathrm{art}}(t-t_0)$ are a Gaussian and its first and second derivatives with identical temporal widths as the apparatus function allowing to account for the coherent artefact, and N is the number of exponentials describing the dynamics of the TA change over time. The reaction schemes sketched in Figure 3 applies to our system and the corresponding differential equation is given in Eq. 9.

$$\frac{\mathrm{d}}{\mathrm{d}t}\begin{pmatrix} [\,{}^1\mathrm{P}_1] \\ [\,{}^3\mathrm{P}_1] \\ [\,{}^2\mathrm{I}_0^{(1)}] \\ [\,{}^2\mathrm{I}_0^{(2)}] \\ [\,{}^1\mathrm{I}_0^{(3)}] \\ [\,{}^1\mathrm{C}_0] \end{pmatrix} = -\begin{pmatrix} k_{\mathrm{ic}}+k_{\mathrm{r}}+k_{\mathrm{isc}}+k_{\mathrm{eT}}^{(1)} & 0 & 0 & 0 & 0 & 0 \\ -k_{\mathrm{isc}} & k_{\mathrm{bisc}} & 0 & 0 & 0 & 0 \\ -k_{\mathrm{eT}}^{(1)} & 0 & k_{\mathrm{beT}}+k_{\mathrm{PT}}^{(1)} & 0 & 0 & 0 \\ 0 & 0 & -k_{\mathrm{PT}}^{(1)} & k_{\mathrm{bHT}}+k_{\mathrm{eT}}^{(2)} & 0 & 0 \\ 0 & 0 & 0 & -k_{\mathrm{eT}}^{(2)} & k_{\mathrm{bH\text{-}T}}+k_{\mathrm{PT}}^{(2)} & 0 \\ 0 & 0 & 0 & 0 & -k_{\mathrm{PT}}^{(2)} & k_{{}^1\mathrm{C}_0} \end{pmatrix}\begin{pmatrix} [\,{}^1\mathrm{P}_1] \\ [\,{}^3\mathrm{P}_1] \\ [\,{}^2\mathrm{I}_0^{(1)}] \\ [\,{}^2\mathrm{I}_0^{(2)}] \\ [\,{}^1\mathrm{I}_0^{(3)}] \\ [\,{}^1\mathrm{C}_0] \end{pmatrix} \tag{9}$$

In cases of unbound PChlide and PChlide bound in the pseudo-ternary complex, PChlide/POR/NADP+, we only observe the photophysical processes but no photochemistry. Thus, Eq. 9 simplifies to Eq. 10.

$$\frac{\mathrm{d}}{\mathrm{d}t}\begin{pmatrix} [\,{}^1\mathrm{P}_1] \\ [\,{}^3\mathrm{P}_1] \end{pmatrix} = -\begin{pmatrix} k_{\mathrm{ic}}+k_{\mathrm{r}}+k_{\mathrm{isc}} & 0 \\ -k_{\mathrm{isc}} & k_{\mathrm{bisc}} \end{pmatrix}\begin{pmatrix} [\,{}^1\mathrm{P}_1] \\ [\,{}^3\mathrm{P}_1] \end{pmatrix} \tag{10}$$

The eigenvalues of the rate constant matrix are given in Eq. 11 to 12. These eigenvalues are absolutely determined by the global fit described above.

$$\kappa_1 = k_{\mathrm{ic}} + k_{\mathrm{r}} + k_{\mathrm{isc}} \tag{11}$$
$$\kappa_2 = k_{\mathrm{bisc}} \tag{12}$$

In this simple model considering the ground state contribution, $\mathrm{S}_{^1\mathrm{P}_0}$, one obtains the following relationship between the SAS, S_i, and the DADS, D_i:

$$\mathrm{S}_{^1\mathrm{P}_1} = \frac{(\mathrm{D}_1 + \mathrm{D}_2)}{c_0} + \mathrm{S}_{^1\mathrm{P}_0} \tag{13}$$
$$\mathrm{S}_{^3\mathrm{P}_1} = \frac{(\kappa_1 - \kappa_2)\mathrm{D}_2}{c_0 \Phi_{^3\mathrm{P}_1} \kappa_1} + \mathrm{S}_{^1\mathrm{P}_0} \tag{14}$$

As can be seen, the only undetermined parameters are c_0 and $\Phi_{^3\mathrm{P}_1}$. However, one can at least find upper or lower bounds by the requirement that the resulting SAS must be positive, and should not show any of the characteristic bands of the other species. In particular the negative peaks from the ground state bleach should disappear in the SAS.

The situation is different in the cases of PChlide bound in the ternary complexes, either PChlide/POR/NADPH or PChlide/POR-C226S/NADPH, where photochemistry can be observed starting from the $\mathrm{S}_{^1\mathrm{P}_1}$ state. Here, in comparison with the non-productive pseudo-ternary complex and assuming that the observed $\mathrm{S}_{^1\mathrm{P}_1}$ lifetime corresponds to the $\mathrm{S}_{^1\mathrm{P}_1}$ lifetime in the ternary complexes, the $\mathrm{S}_{^1\mathrm{P}_1}$ state is quenched from 3.9 ns and 4.4 ns to 650 ps and 760 ps in case of wild type and the C226S variant, respectively. Then, according $\Phi_{^2\mathrm{I}_0^{(1)}} = 1 - \tau/\tau_0$, where τ_0 and τ are the $\mathrm{S}_{^1\mathrm{P}_1}$ lifetimes of the unreactive pseudo- and corresponding reactive ternary complexes, respectively, the quantum yield for the newly formed intermediate, $^2\mathrm{I}_0^{(1)}$, is 83% in both cases. Taking into account the intersystem crossing rates, k_{isc}, determined in the pseudo-ternary complexes, one would expect triplet yields of *ca.* 8% and 9% in case of the wild type and the C226S variant, respectively. However, we do not observe the formation of the triplet in the case of the productive ternary complexes, which led us conclude that in the ternary complexes k_{isc} must be negligible. Thus, Eq. 9 simplifies to Eq. 15 in the case of the ternary complexes.

$$\frac{\mathrm{d}}{\mathrm{d}t}\begin{pmatrix}[\,^1\mathrm{P}_1]\\ [\,^2\mathrm{I}_0^{(1)}]\\ [\,^2\mathrm{I}_0^{(2)}]\\ [\,^1\mathrm{I}_0^{(3)}]\\ [\,^1\mathrm{C}_0]\end{pmatrix} = -\begin{pmatrix} k_{\mathrm{ic}} + k_{\mathrm{r}} + k_{\mathrm{eT}}^{(1)} & 0 & 0 & 0 & 0\\ -k_{\mathrm{eT}}^{(1)} & k_{\mathrm{beT}} + k_{\mathrm{PT}}^{(1)} & 0 & 0 & 0\\ 0 & -k_{\mathrm{PT}}^{(1)} & k_{\mathrm{bHT}} + k_{\mathrm{eT}}^{(2)} & 0 & 0\\ 0 & 0 & -k_{\mathrm{eT}}^{(2)} & k_{\mathrm{bH^-T}} + k_{\mathrm{PT}}^{(2)} & 0\\ 0 & 0 & 0 & -k_{\mathrm{PT}}^{(2)} & k_{^1\mathrm{C}_0}\end{pmatrix}\begin{pmatrix}[\,^1\mathrm{P}_1]\\ [\,^2\mathrm{I}_0^{(1)}]\\ [\,^2\mathrm{I}_0^{(2)}]\\ [\,^1\mathrm{I}_0^{(3)}]\\ [\,^1\mathrm{C}_0]\end{pmatrix} \tag{15}$$

The eigenvalues of the rate constant matrix are given in Eq. 16-20. These eigenvalues are absolutely determined by the global fit described above.

$$\kappa_1 = k_{\mathrm{ic}} + k_{\mathrm{r}} + k_{\mathrm{eT}}^{(1)} \tag{16}$$
$$\kappa_2 = k_{\mathrm{beT}} + k_{\mathrm{PT}}^{(1)} \tag{17}$$
$$\kappa_3 = k_{\mathrm{bHT}} + k_{\mathrm{eT}}^{(2)} \tag{18}$$
$$\kappa_4 = k_{\mathrm{bH^-T}} + k_{\mathrm{PT}}^{(2)} \tag{19}$$
$$\kappa_5 = k_{^1\mathrm{C}_0} \tag{20}$$

Correspondingly, Eq. 15 can also be expressed in terms of the eigenvalues which are determined by the global exponential fit on data matrix yielding Eq. 21.

$$\frac{\mathrm{d}}{\mathrm{d}t}\begin{pmatrix}[\,^1\mathrm{P}_1]\\ [\,^2\mathrm{I}_0^{(1)}]\\ [\,^2\mathrm{I}_0^{(2)}]\\ [\,^1\mathrm{I}_0^{(3)}]\\ [\,^1\mathrm{C}_0]\end{pmatrix} = -\begin{pmatrix} \kappa_1 & 0 & 0 & 0 & 0\\ -\Phi_{^2\mathrm{I}_0^{(1)}}\kappa_1 & \kappa_2 & 0 & 0 & 0\\ 0 & -\Phi_{^2\mathrm{I}_0^{(2)}}\kappa_2 & \kappa_3 & 0 & 0\\ 0 & 0 & -\Phi_{^1\mathrm{I}_0^{(3)}}\kappa_3 & \kappa_4 & 0\\ 0 & 0 & 0 & -\Phi_{^1\mathrm{C}_0}\kappa_4 & k_5\end{pmatrix}\begin{pmatrix}[\,^1\mathrm{P}_1]\\ [\,^2\mathrm{I}_0^{(1)}]\\ [\,^2\mathrm{I}_0^{(2)}]\\ [\,^1\mathrm{I}_0^{(3)}]\\ [\,^1\mathrm{C}_0]\end{pmatrix} \tag{21}$$

The model leads to the following relationship between the SAS, S_i, and the DADS, D_i:

$$\mathrm{S}_{^1\mathrm{P}_1} = \frac{(\mathrm{D}_1 + \mathrm{D}_2 + \mathrm{D}_3 + \mathrm{D}_4 + \mathrm{D}_5)}{c_0} + \mathrm{S}_{^1\mathrm{P}_0} \tag{22}$$
$$\mathrm{S}_{^2\mathrm{I}_0^{(1)}} = \frac{(\kappa_1 - \kappa_2)\mathrm{D}_2 + (\kappa_1 - \kappa_3)\mathrm{D}_3 + (\kappa_1 - \kappa_4)\mathrm{D}_4 + (\kappa_1 - \kappa_5)\mathrm{D}_5}{c_0 \Phi_{^2\mathrm{I}_0^{(1)}} \kappa_1} + \mathrm{S}_{^1\mathrm{P}_0} \tag{23}$$

$$S_{^2I_0^{(2)}} = \frac{(\kappa_1-\kappa_3)(\kappa_2-\kappa_3)D_3+(\kappa_1-\kappa_4)(\kappa_2-\kappa_4)D_4+(\kappa_1-\kappa_5)(\kappa_2-\kappa_5)D_5}{c_0\Phi_{^2I_0^{(1)}}\kappa_1\Phi_{^2I_0^{(2)}}\kappa_2} + S_{^1P_0} \qquad (24)$$

$$S_{^1I_0^{(3)}} = \frac{(\kappa_1-\kappa_4)(\kappa_2-\kappa_4)(\kappa_3-\kappa_4)D_4+(\kappa_1-\kappa_5)(\kappa_2-\kappa_5)(\kappa_3-\kappa_5)D_5}{c_0\Phi_{^2I_0^{(1)}}\kappa_1\Phi_{^2I_0^{(2)}}\kappa_2\Phi_{^1I_0^{(3)}}\kappa_3} + S_{^1P_0} \qquad (25)$$

$$S_{^1C_0} = \frac{(\kappa_1-\kappa_5)(\kappa_2-\kappa_5)(\kappa_3-\kappa_5)(\kappa_4-\kappa_5)D_5}{c_0\Phi_{^2I_0^{(1)}}\kappa_1\Phi_{^2I_0^{(2)}}\kappa_2\Phi_{^1I_0^{(3)}}\kappa_3\Phi_{^1C_0}\kappa_4} + S_{^1P_0} \qquad (26)$$

Here, c_0 is the contribution of the ground state spectrum and the Φ_i are the yields for the conversion from state i to the subsequent state $i+1$ as given by Eq. 27.

$$\Phi_i = \frac{k_{i\to i+1}}{\kappa_i} \qquad (27)$$

As can be seen, the only undetermined parameters are c_0, $\Phi_{^2I_0^{(2)}}$, $\Phi_{^1I_0^{(3)}}$, and $\Phi_{^1C_0}$. However, again, one can at least find upper or lower bounds by the requirement that the resulting SAS must be positive, and should not show any of the characteristic bands of the other species. In particular the negative peaks from the ground state bleach should disappear in the SAS. In Figures S13 to S16 the determination of the individual parameters is shown for each individual case.

Correction of free PChlide contributions.
It is generally accepted that PChlide-binding to POR occurs only in the presence of NADPH. Thus, 100% binding could not be achieved due to a significantly reduced binding affinity for $NADP^+$ resulting in reduced PChlide-binding.[17] As an unavoidable consequence, in all time-resolved data the signals of unbound PChlide needed to be accounted for. In the case of the pseudo-ternary complex, PChlide/POR/$NADP^+$, only *ca.* 50% of bound PChlide could be obtained due to the poor binding affinity of $NADP^+$. Thus, the TA data included both the dynamics of bound and unbound PChlide. Fortunately, two distinct DADS could be obtained for either the bound or unbound PChlide dynamics of the $^3P_1$ state allowing for a separate analysis. The $^3P_1$ decay shows a bi-exponential behavior with lifetimes of 4.6 and 19 μs. The faster lifetime corresponds to unbound PChlide (τ_2 in Figure S6 **C**) which is evidenced by its blue shifted TA features compared to the DADS with the 19 μs lifetime (Figure S8 **C**). However, the singlet lifetime of (un)bound PChlide is almost identical resulting in a single DADS for both situations. Since the sample consisted of a *ca.* one to one mixture of (un)bound PChlide, the contributions of unbound PChlide were accounted for as follows. First the DADS of the pure $^1P_1$ decay obtained from data on PChlide in buffer was scaled on top of the DADS with the mixed dynamics. Then *ca.* 50% of the scaled DADS were subtracted.

Discussion about the PChlide photophysics found in literature.
Since PChlide acts as a photoreceptor, the POR-catalyzed reaction also depends on the excited state dynamics of the substrate itself. The excited state dynamics of (un)bound PChlide were studied by various time-resolved spectroscopy techniques and different – branched and sequential – models were proposed, all starting from the excited singlet state. These models include the formation of a so called S_{ICT} state, which is defined differently in the two models. In the branched model, it is proposed to be an intramolecular charge-transfer state formed from the excited singlet state, that is suggested to induce an electron-deficient site at the C_{17}-C_{18} double bond of PChlide and finally facilitate the selective reduction of this bond. In the sequential model, it is proposed to be of internal charge transfer character of the S_1 state, which was initially proposed by.[18] The PChlide triplet state was also observed in both models, however, it was proposed to be unimportant in the photoreduction.[19,20] According to the sequential model, the excited singlet state decays independently of the solvent polarity into the S_{ICT} state with a lifetime of *ca.* 10 ps, followed by its relaxation into the solvated S_{ICT} with a lifetime of *ca.* 360 ps in aqueous buffer.[21,22] Subsequently, the solvated S_{ICT} decays into the triplet state with a lifetime of 2.6 ns,[20] which finally decays back into the ground state with a lifetime of either 110 μs in aqueous buffer or 2 μs in methanol.[23] These states and their corresponding dynamics were proposed based on time-resolved absorption in the visible and the infrared as well as on emission spectroscopy data.[20,24,25] The data were analyzed by fitting a sequential model (one state converting entirely into the following excluding ground state recovery) yielding so called evolution associated difference spectra (EADS).[20] Interestingly, the absorption spectra of the states, S_1, S_{ICT}, and solvated S_{ICT} have all the same spectral features and are only distinguished by differing lifetimes. In contrast, data are interpreted differently according to the branched model. In this case, the authors state that from the Franck-Condon region on the one hand the S_1 state is formed with a lifetime of 3.5 ps and on the other hand, if the solvent environment is polar, the S_{ICT} is formed with a lifetime of *ca.* 27 ps. The proposed S_{ICT} decays with a lifetime of 200 ps into the ground state S_0, whereas the S_1 decays either into the ground state *via* fluorescence or into the triplet state with a lifetime of 3.4 ns.[19,26–28] Here, the S_{ICT} is characterized by an additional absorption band between 670 and 700 nm compared to the S_1 state absorption,[19] which could only be observed in polar solvents, *e.g.* methanol or acetonitrile.[26,29,30] The S_{ICT} is proposed to result from the electron-withdrawing effect of the C13$_1$=O carbonyl group in ring E of PChlide, which is directly attached to the π–electron conjugation path of the porphyrin macrocycle.[19,26–30] However, most recently the 'reactive' S_{ICT} leading directly to POR-photochemistry was proposed to result from an electron-withdrawing effect on the C_{17}-C_{18} double bond formed by excited state H-bonding interactions between the OH group of a conserved tyrosine residue within the PChlide-binding pocket and the carbonyl group of the propionic acid side chain at the C_{17} position of PChlide.[24] In both models, the (solvated) S_{ICT} state is proposed to be of importance in POR photo-catalysis. However, the presented data, especially the proposed dynamics of the S_{ICT} state, the spectral characteristics as well as the nature of the triplet precursor, differ significantly. Since a detailed understanding of the PChlide photophysics is essential for the interpretation of the photochemistry catalyzed by POR, we re-investigated the light-induced dynamics obtained for the unbound PChlide and when bound in the non-productive pseudo-ternary complex, PChlide/POR/$NADP^+$. The results are presented in the following paragraph.

Initially, we investigated the unbound substrate PChlide. The stationary absorption spectrum (Figure S2 **A**), especially the Q-band at *ca.* 625 nm, has a strong dependence on the solvent polarity (Figure S1) in agreement with previous reports.[26,31] For instance, the

Q-band absorption is blue shifted in acetonitrile (ACN) compared to aqueous buffer from 630 to 623 nm accompanied by an increase of the extinction coefficient by 21%. Initially, after excitation in the Soret band at *ca.* 450 nm a broad transient absorption (TA) signal, $\Delta A(t,\lambda)$, is observed with positive excited state absorption (ESA) features peaking at 368(372), 471(481), 593(597), 653(664), 721(724), 754(761), and 780(784) nm and negative ground state bleach (GSB) features peaking at 437(442) and 625(633) nm (Figures S3 and S4, these and following values correspond to data obtained when dissolved in ACN(Buffer)). In the first picosecond, the GSB increases and, *e.g.*, the ESA band at 471(481) nm narrows and decreases with a lifetime of *ca.* 112(142) fs. These spectral changes are not observed, when exciting into the Q-band at *ca.* 630 nm indicating the internal conversion (IC) of higher excited singlet states of PChlide, $^1P_4$, into the first excited singlet state, $^1P_1$ (Figures S3-4 **B1-2**). This disagrees with the previously found value of *ca.* 700 fs.[20] In the following 50 ps the overall shape of this difference absorption spectrum changes slightly in terms of small spectral shifts as well as narrowing/broadening of some ESA features with a lifetime(s) of 16(6 and 53) ps indicating vibrational and solvent relaxation until the thermally equilibrated $^1P_1$ state is formed (Figures S3-4 **D1-2**). This agrees with the previously described solvation dynamics observed *via* spectral shifts in time-resolved emission spectra of PChlide on a similar time scale.[25] After all relaxation processes have settled, significant TA changes are observed indicating changes in the population of electronic states (Figure S2 **B**), which are assigned to represent the conversion from the $^1P_1$ to either the triplet, $^3P_1$, or to the ground state, $^1P_0$. The equivalent dynamics can simply be described by two rate constants with 4 and 250 ns (Figure S2 **B**). The corresponding Decay Associated Difference Spectra (DADS) show, respectively, the conversion from $^1P_1$ to $^3P_1$ and $^1P_0$, and from $^3P_1$ to $^1P_0$ (The decay of $^1P_1$ with a lifetime of 4.3 ns is also confirmed by spectrally- and time-resolved emission data shown in Figures S2 **A** and S5). Since only two rate constants are found, a standard photophysical model with an excited singlet and a triplet state appears most likely to describe the observed data (Figure 3 in the main article). Based on this model we determined the Species Associated Spectra (SAS) for the PChlide excited singlet ($^1P_n \leftarrow {}^1P_1$) and the triplet state ($^3P_n \leftarrow {}^3P_1$) (Figure 4 **A** in the main article), with a triplet yield from $^1P_1$ of $\Phi(^3P_1, \text{ACN}) \approx 0.5$ (For details of the modelling procedure see above). In contrast to the $^1P_1$, the $^3P_1$ is mainly quenched by molecular oxygen (O_2) seen by an increased lifetime from 250 ns to 170 μs, when O_2 is removed from the sample (Figure S2 **C-D**). Thus, the intrinsic back intersystem crossing rate $k_{\text{bisc}} = (170\ \mu s)^{-1} = 5.88 \cdot 10^3\ s^{-1}$ together with the concentration of O_2, $[O_2]$ (c = 2.4 mM in ACN[32]), gives a O_2-quenching rate to $k_q = 1.7 \cdot 10^9\ M^{-1}s^{-1}$. In aqueous buffer (containing 40 Vol% glycerol) very similar SAS are obtained, but the $^3P_1$ lifetime is increased to 4.8 μs due to slower diffusion of O_2 reducing the quenching efficiency (Figures S6). The $^3P_1$ yield is determined to $\Phi(^3P_1, \text{buffer}) \approx 0.55$.

In contrast to previous reports, we do not observe the formation of a distinct charge-separated S_{ICT} state based on the following observations. The formation of a charge-separated state across the porphyrin macrocycle is expected to result in the formation of distinct different absorption bands, which are not observed in the present study although polar solvents were used (Figures S3-4). This is further emphasized by the fact, that the observed slight changes in the difference absorption spectrum during the first 50 ps after photo-excitation, *i.e.* small spectral shifts as well as narrowing/broadening of some ESA features with a lifetime(s) of 16(6 and 53) ps when dissolved in ACN(Buffer), occur on time-scales typical for vibrational and solvent relaxation until the thermally equilibrated S_1 state is formed. The observation of only two rate constants on longer time-scales led us conclude, that a standard photophysical model with an excited singlet and a triplet state (Figure S2) appears most likely to describe the observed data. Also, considering the presence of a carbonyl group, $C13_1$=O, explains well the observed high triplet quantum yield due to available spin-orbit coupling in the molecule, *i.e.* purely $\pi \rightarrow \pi^*$ in S_1 and $n \rightarrow \pi^*$ transition character in T_1 and T_2, as in line with the El-Sayed rules.[33,34] In addition, the highly conjugated and planar nature of the PChlide molecule lacks molecular moieties necessary for stabilising a concrete ICT state formed from the locally excited singlet state. Thus, the formation of a charge-separated S_{ICT} state in such a molecule seems to be unlikely anyway. However, it is likely that the $S_1 \leftarrow S_0$ transition is of charge-transfer character, because PChlide consists of a carbonyl group at C13. Thus, we performed QM calculations of the excited states of PChlide at a qualitative level. No intramolecular charge-transfer state below the locally excited singlet state is found. Only for the transition from S_0 to the lowest S_1 state a small charge transfer character is found as electron density is moved to a small extent towards the $C13_1$=O carbonyl group of the isocyclic ring (see Figures S19-20). This finding supports further the importance of this ring as reported previously.[3,25] In a recent work the propionic acid side chain at the C17 position is suggested to form an interaction with the highly conserved active site residue Tyr193 resulting in an electron withdrawing effect.[24] However, the calculations do not support this. Nevertheless, the importance of this moiety remains to be determined, since some analogues containing changes at the C17 position remain inactive (*e.g.* chlorophyll c), and that conservative mutagenesis of Tyr-193 to Phe-193 results in loss of activity and impaired photochemistry.[3,24]

We then investigated the non-productive pseudo-ternary complex, PChlide/POR/$NADP^+$, in order to account for H-bonding interactions between PChlide and the protein pocket. Due to binding to the protein pocket the maxima of the ground state absorption spectrum of PChlide are red shifted compared to the unbound situation (see Figure S8 **A**). Initially, after photoexcitation, the ESA shows slight spectral red shifts (ESA: 598(597), 730(724), and 762(761); GSA: 444(442) and 640(633) nm; in complex(Buffer)) in accordance to the red shift already seen in the GS absorption. Otherwise the TA data resemble those data obtained from unbound PChlide in aqueous buffer (see Figures S7 **B**). On the first 50 ps, prior to the thermalization of the $^1P_1$ the $^1P_4$ to $^1P_1$ conversion followed by vibrational and solvent relaxation is observed as seen in slight changes of the overall shape of the TA spectrum with lifetimes of 116 fs and 4.2 ps, respectively (Figure S8 **B1-2, D1-2**). On the ns timescale, the $^1P_1$ decays with an identical lifetime as in the unbound situation (Figures S8, S9). The $^3P_1$ lifetime is with 19 μs (Figure S8 **C**) *ca.* 4 times longer compared to unbound PChlide in buffer (τ = 4.8 μs). Without O_2 the $^3P_1$ lifetime prolongs to 73 μs (data not shown) yielding $k_{\text{bisc}} = (73\ \mu s)^{-1} = 1.4 \cdot 10^4\ s^{-1}$. Together with the concentration of O_2 (c = 0.25 mM in H_2O[35]) the k_q^{O2} yields $1.6 \cdot 10^8\ M^{-1}s^{-1}$. In comparison, the k_q^{O2} of unbound PChlide in buffer is with $8.3 \cdot 10^8\ M^{-1}s^{-1}$ 5.2 times larger (without O_2 the lifetime prolongs from 4.8 μs to *ca.* 1 ms yielding $k_{\text{bisc}} = 1\ (ms)^{-1} = 1.0 \cdot 10^3\ s^{-1}$). This demonstrates a protective role of POR against reactions with O_2 within the lifetime of the $^3P_1$, thus, avoiding the formation of reactive oxygen species (ROS) as long as the individual intermediates within the chlorophyll biosynthesis cannot be found unbound *in vivo* and are rather transferred from enzyme to enzyme. The modelling of the SAS yields almost identical SAS for $^1P_1$ and $^3P_1$ compared to unbound PChlide (Figure 4 **B** in the main article), with a similar $^3P_1$ yield of $\Phi(^3P_1, \text{PChlide/POR/NADP}^+) \approx 0.55$.

Discussion about physical feasibility of the dramatic quenching of the PChlide excited singlet in the productive ternary complex POR/NADPH/PChlide.

In general, the decay of a S_1 state is defined by the sum of all standard photo-physical processes, namely internal conversion, k_{ic}, radiative decay (fluorescence), k_{r}, and intersystem-crossing, k_{isc}, plus additional processes such as energy transfer or electron transfer combined in k_{q} (Eq. 28).

$$k_{\mathrm{S_1}} = k_{\mathrm{ic}} + k_{\mathrm{r}} + k_{\mathrm{isc}} + k_{\mathrm{q}} \quad (28)$$

An increased k_{r} can be excluded, since there is no emission in the ternary complex. An increased k_{ic} due to promoting motions can be excluded, since we observe the formation of a new electronic species and not an increased recovery into the ground state. An increased k_{isc} can be excluded, since we do not observe the formation of a triplet at all. Energy transfer can be excluded, since there is no acceptor present in the sample. Therefore, electron transfer is the most probable mechanism causing quenching of the excited singlet state.

Discussion about known species spectra from light-independent POR.

Nomata *et al*. propose that similar species are involved in the PChlide reduction of a light-independent POR and provide UV/Vis absorption spectra for each intermediate.[36] However, due to the close proximity to an additional Fe-S-cluster, which acts as the electron source during the PChlide reduction, PChlide and formed intermediates experience totally different Coulombic interactions. Thus, a direct comparison to the situation in light-dependent POR cannot be made. Furthermore, those spectra were recorded at 5°C. With this in mind, state 1 in Nomata *et al.,* which is assigned to a PChlide radical anion is not that different compared to our spectrum of the radical anion. The spectrum shown for state 2 in Nomata *et al*., however, corresponds to the Chlide anion, *e.g.* prominent and intense absorption band around 700 nm, from our and other previous studies (Heyes *et al.* 2009[37]), which we assign as intermediate 3. Nomata *et al.* assign this spectrum to the neutral radical, which considering our calculations (Fig. S17) and known data from other studies, *e.g.* Heyes *et al.* 2009, questions their assignment. Finally, the overall shape of state 3 from Nomata *et al.*, which is assigned to Chlide anion, shares similar absorption features to our spectrum of the neutral radical (intermediate 2).

Figure S1. Stationary absorption spectra of PChlide in various solvents. **A**: Decreasing solvent polarity from red to blue in buffer, ethanol (EtOH), ethanol/acetonitrile 1 to 1 mixture (EtOH/ACN), acetonitrile (ACN), ethylacetate (EtAc), and di-ethylether (EtOEt), respectively. **B**: Comparison between solvation in buffer (black) and ACN (blue).

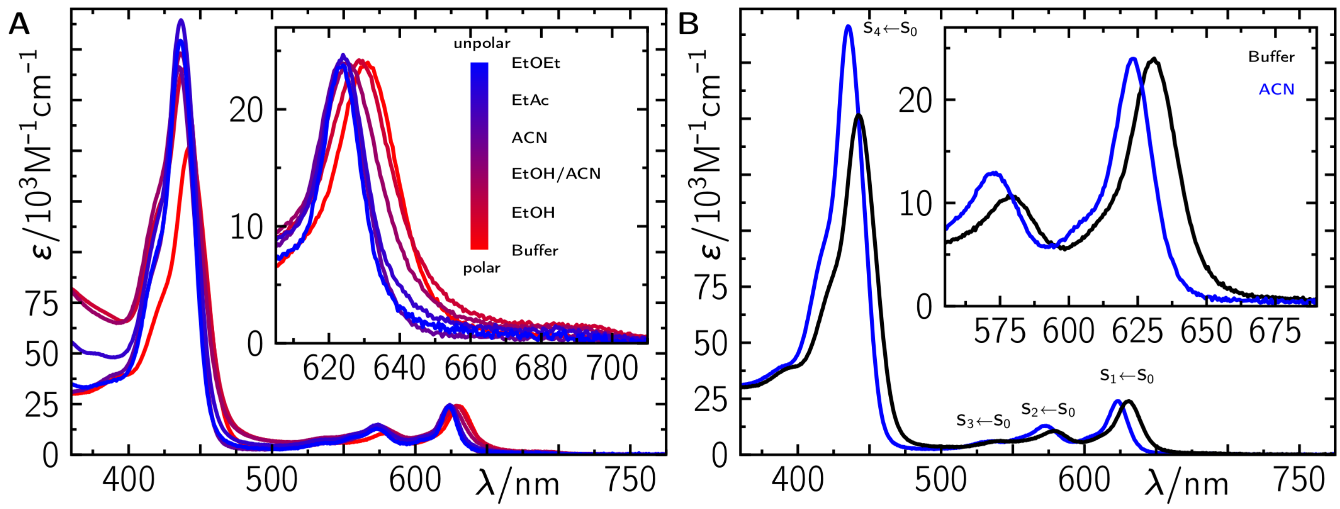

Figure S2. Photophysical characterisation of unbound PChlide in ACN. **A**: Stationary absorption (black and zoom of Q-band blue) and time-resolved emission spectra (λ_{exc} = 450 nm, red). **B**: Time-resolved difference absorption spectra after excitation at λ_{exc} = 450 nm. **C**: Selected time traces obtained from samples under (an)aerobic conditions. **D**: Decay Associated Difference Spectra (DADS) from global bi-exponential fit on data in **B**.

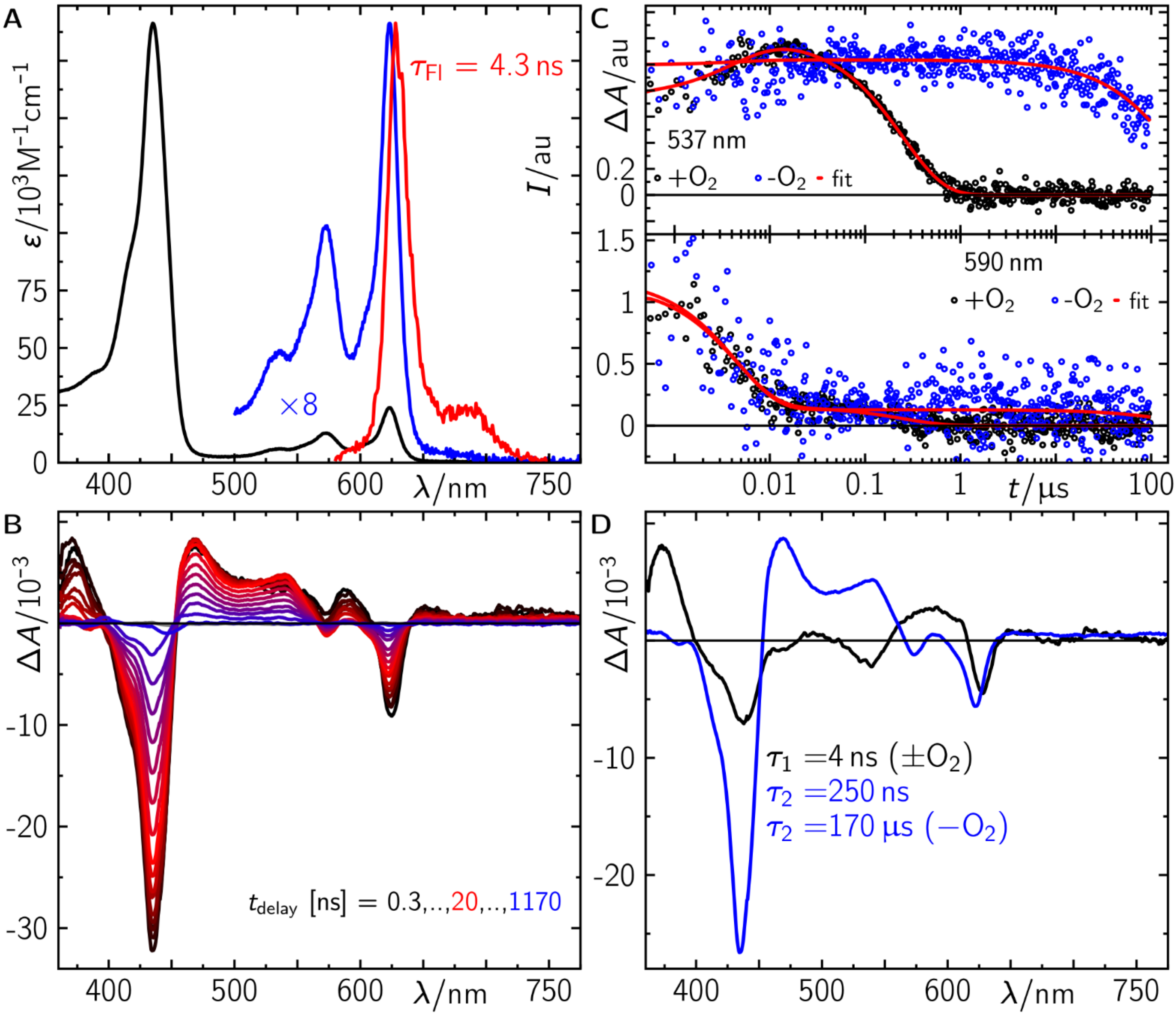

Figure S3. Time-resolved difference absorption spectra of unbound PChlide dissolved in ACN recorded in the first 100 ps after photo-excitation into the Soret band (λ_{exc} = 450 nm, $^1P_4 \leftarrow {}^1P_0$ transition). **A** and **C**: Selected TA spectra at time delays up to 1 and 43 ps, respectively. **B1-2**: Changes of the band areas of the

excited state absorption signal at *ca*. 436 nm and *ca.* 464 nm, respectively (black circles), obtained from fitting Gaussians to each difference absorption spectrum (an exemplary fit at t = 50 fs is shown in the inset of panel **A**). **D1-2**: Changes of the band position and width, respectively, of the excited state absorption signal at *ca*. 455 nm (black circles) obtained from fitting Gaussians to each difference absorption spectrum at different delay times (an exemplary fit at t = 1 ps is shown in the inset of panel **C**). The red lines in panels **B1-2** and **D1-2** represent global fits to all obtained parameters of the Gaussians describing the difference spectra (for further details see experimental section).

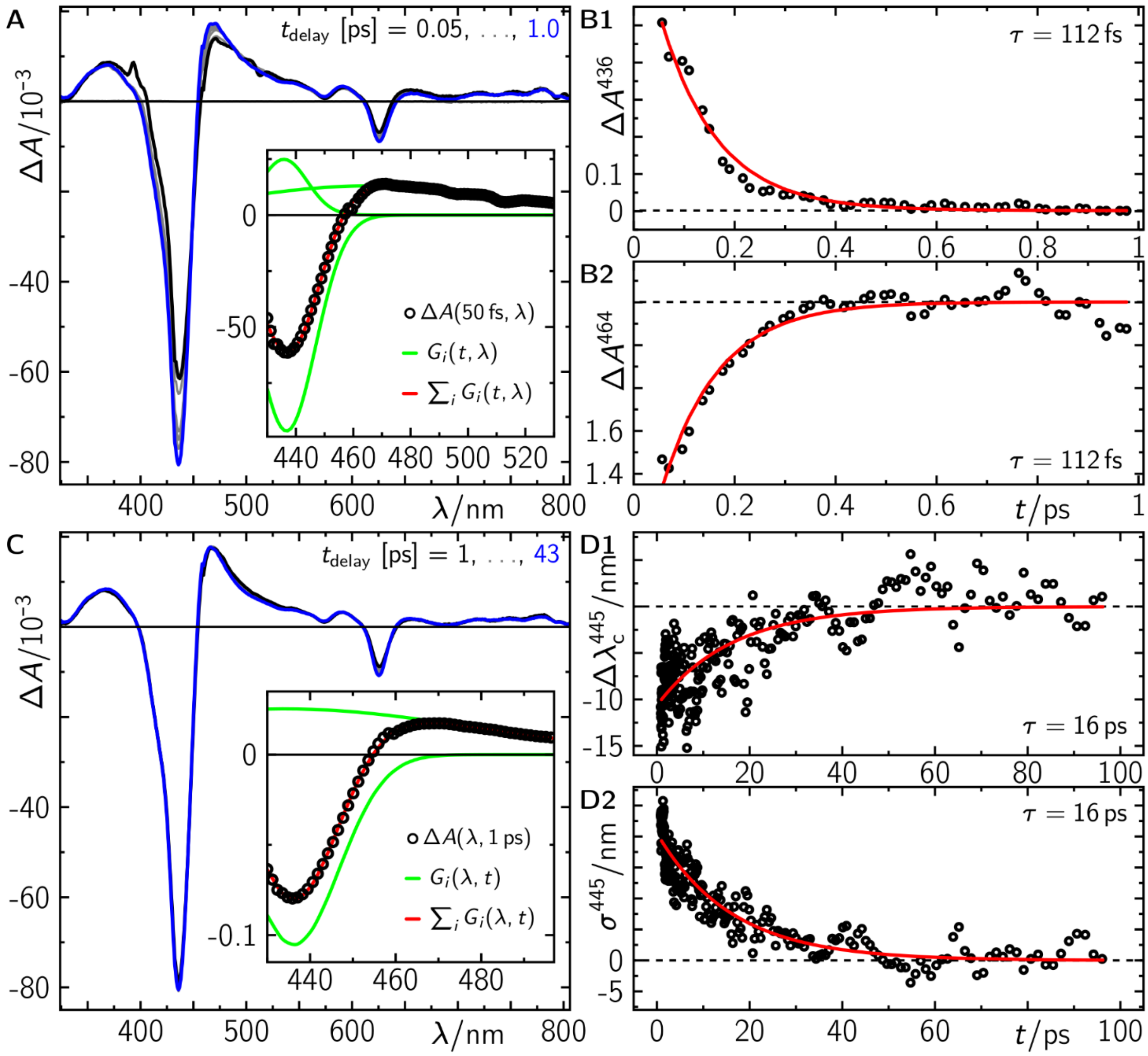

Figure S4. Time-resolved difference absorption spectra of unbound PChlide dissolved in buffer recorded in the first 100 ps after photo-excitation into the Soret band (λ_{exc} = 450 nm, $^{1}P_{4} \leftarrow {}^{1}P_{0}$ transition). **A** and **C**: Selected TA spectra at time delays up to 1 and 19 ps, respectively. **B1-2**: Changes of the band position and area, respectively, of the excited state absorption signal at *ca*. 465 nm (black circles) obtained from fitting Gaussians to each difference absorption spectrum (an

exemplary fit at t = 103 fs is shown in the inset of panel **A**). The grey circles show the corresponding time dependence when excited into the Q-band (λ_{exc} = 624 nm, $^1P_1 \leftarrow {}^1P_0$ transition) indicating no relaxation from the $^1P_4$ into the $^1P_1$. **D1-2**: Changes of the band position and width, respectively, of the excited state absorption signal at *ca*. 595 nm (black circles) obtained from fitting Gaussians to each difference absorption spectrum at different delay times (an exemplary fit at t = 1 ps is shown in the inset of panel **C**). The red lines in panels **B1-2** and **D1-2** represent global fits to all obtained parameters of the difference spectra describing Gaussians (for further details see experimental section).

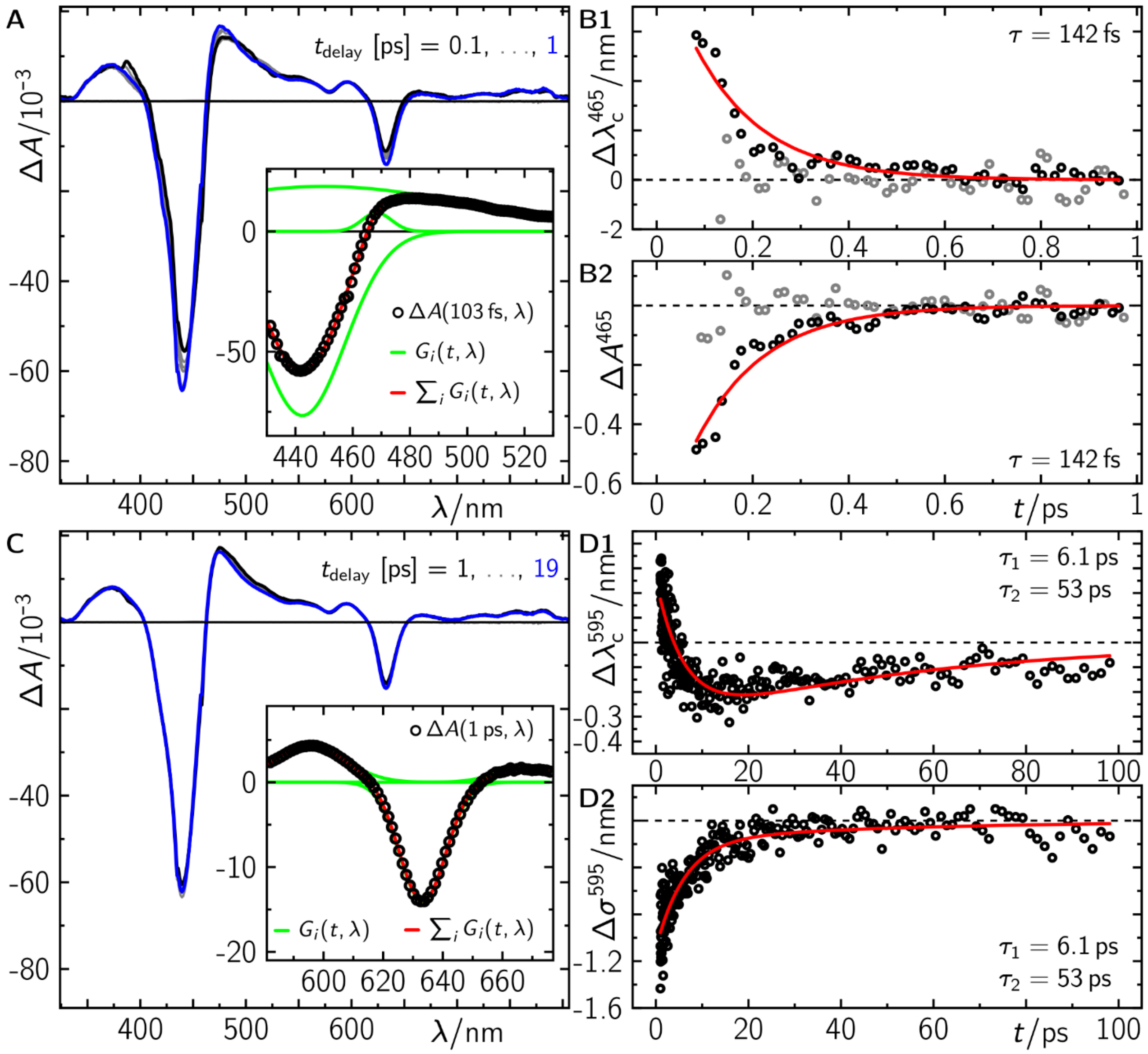

Figure S5. Spectrally and temporally resolved emission spectra of PChlide in ACN (**A**) and in Buffer (**B**) excited at 450 nm. In each panel, the left-hand false colour plot represents the raw data. The right-hand time trace represents the summed data (black circles), the corresponding global fits (red lines) and the resulting residuals (weighted by the square-root of the counted events at each time-point accounting for Poissonian statistics) in the spectral range as indicated by

the dashed black rectangle on top of the false colour plot. The lifetimes are 4.3 ns and 4.0 ns, respectively. The grey dashed rectangle indicates a spectral region, where artificial signals were counted due to overmodulation on the data image caused by the intense laser. This was replaced by the fit during the global fitting procedure as described elsewhere.[8]

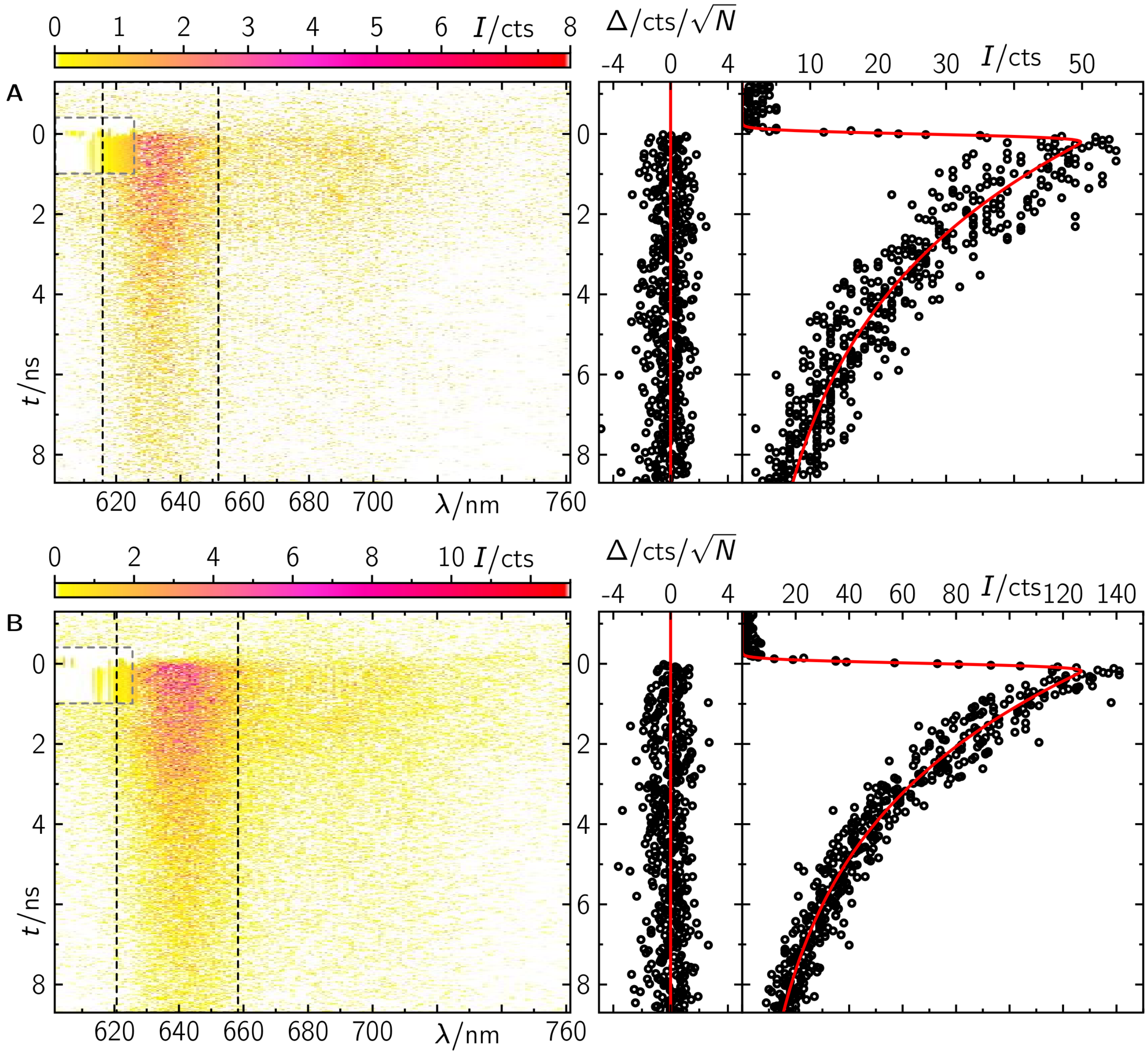

Figure S6. Photophysical characterisation of unbound PChlide in buffer solution. **A**: Stationary absorption (black and zoom of Q-band blue) and time-resolved emission spectra recorded with a streak camera (λ_{exc} = 450 nm, red). **B**: Time-resolved difference absorption spectra after excitation at λ_{exc} = 450 nm. **C**: Decay Associated Difference Spectra (DADS) from global bi-exponential fit on data in **B**. **D**: Species Associated Spectra (SAS) based on the model described in the main text.

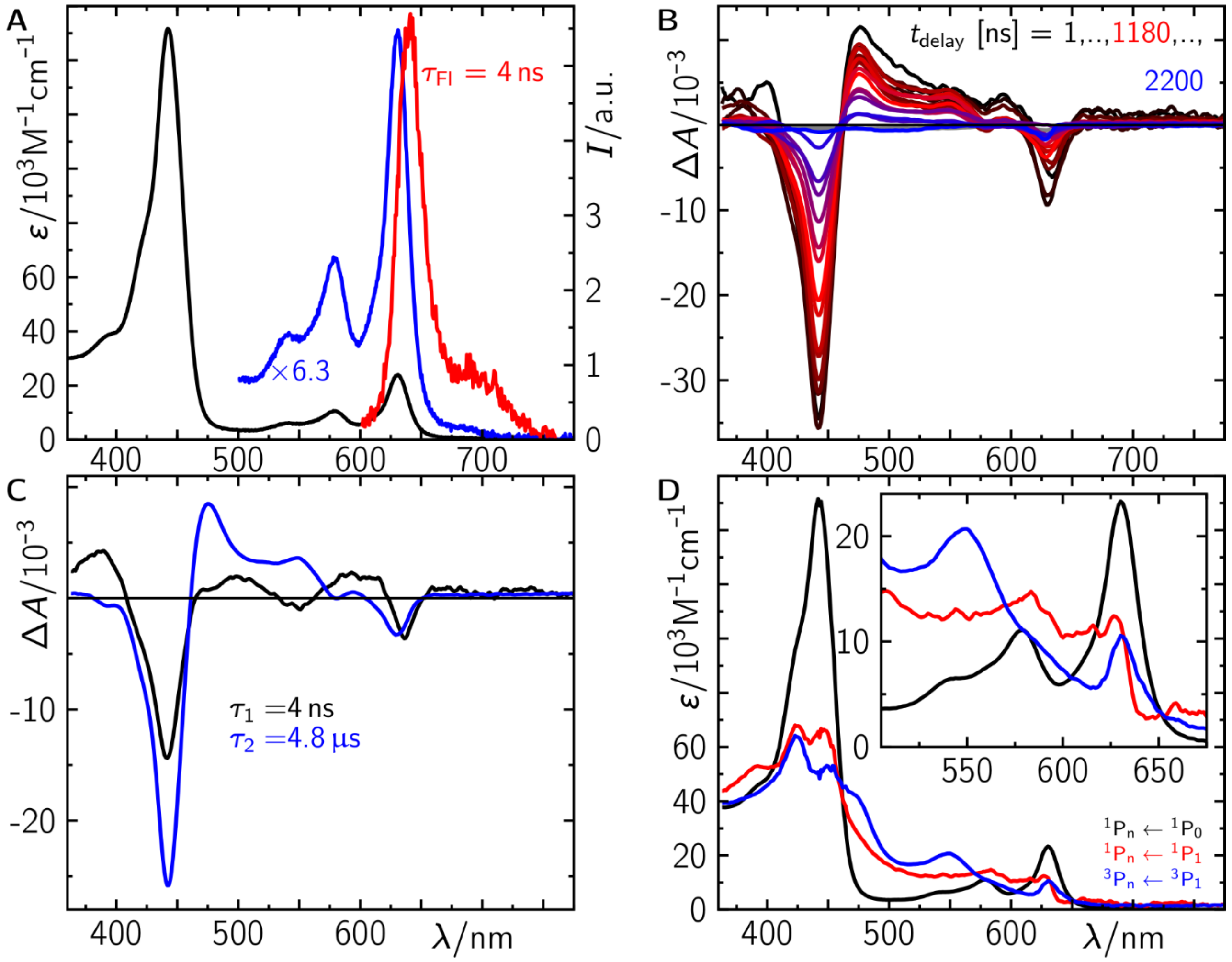

Figure S7. Time-resolved difference absorption spectra of POR-bound PChlide in the pseudoternary complex (non-productive PChlide/POR/NADP$^+$-complex) recorded in the first 50 ps after photo-excitation into the Soret band (λ_{exc} = 450 nm, $^1P_4 \leftarrow {}^1P_0$ transition). **A** and **C**: Selected TA spectra at time delays up to 0.5 and 19 ps, respectively. **B1-2**: Changes of the band width and area of the excited state absorption signal at *ca*. 600 nm and 650 nm (black circles), respectively, obtained from fitting Gaussians to each difference absorption spectrum at different delay times (an exemplary fit at *t* = 78 fs is shown in the inset of panel **A**). **D1-2**: Changes of the band position and width, respectively, of the excited state absorption signal at *ca*. 650 nm (black circles) obtained from fitting Gaussians to each

difference absorption spectrum (an exemplary fit at *t* = 1 ps is shown in the inset of panel **C**). The red lines in panels **B1-2** and **D1-2** represent global fits to all obtained parameters of the difference spectra describing Gaussians (for further details see experimental section).

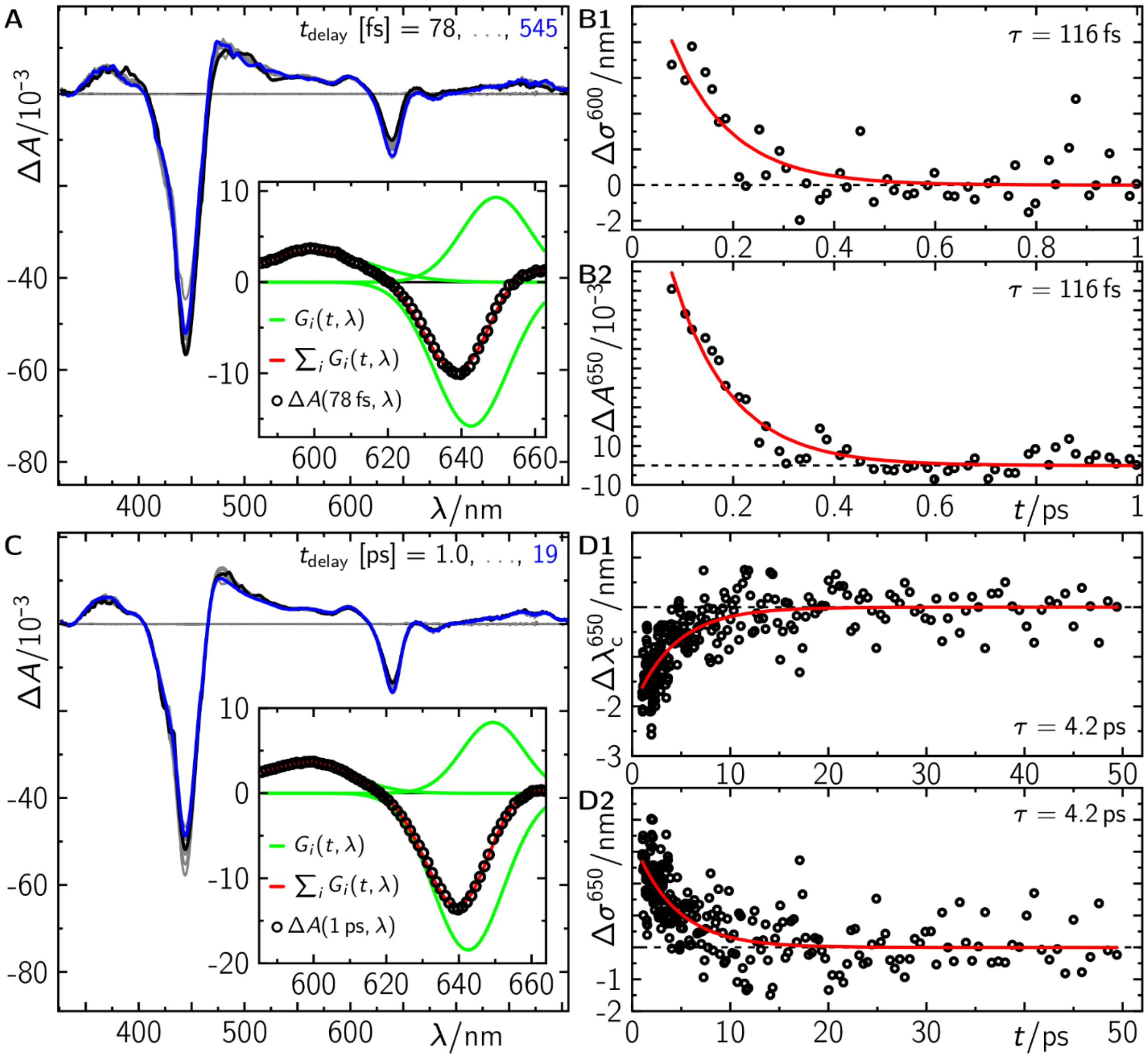

Figure S8. Photophysical characterisation of POR-bound PChlide in the pseudo-ternary complex (non-productive PChlide/POR/NADP$^+$-complex). **A**: Stationary absorption spectra of unbound (black) and bound (blue) PChlide, and time-resolved emission spectra recorded with a streak camera (λ_{exc} = 450 nm, red). **B**: Time-resolved difference absorption spectra after excitation at λ_{exc} = 635 nm. **C**: Decay Associated Difference Spectra (DADS) from a global tri-exponential fit on data in **B**.

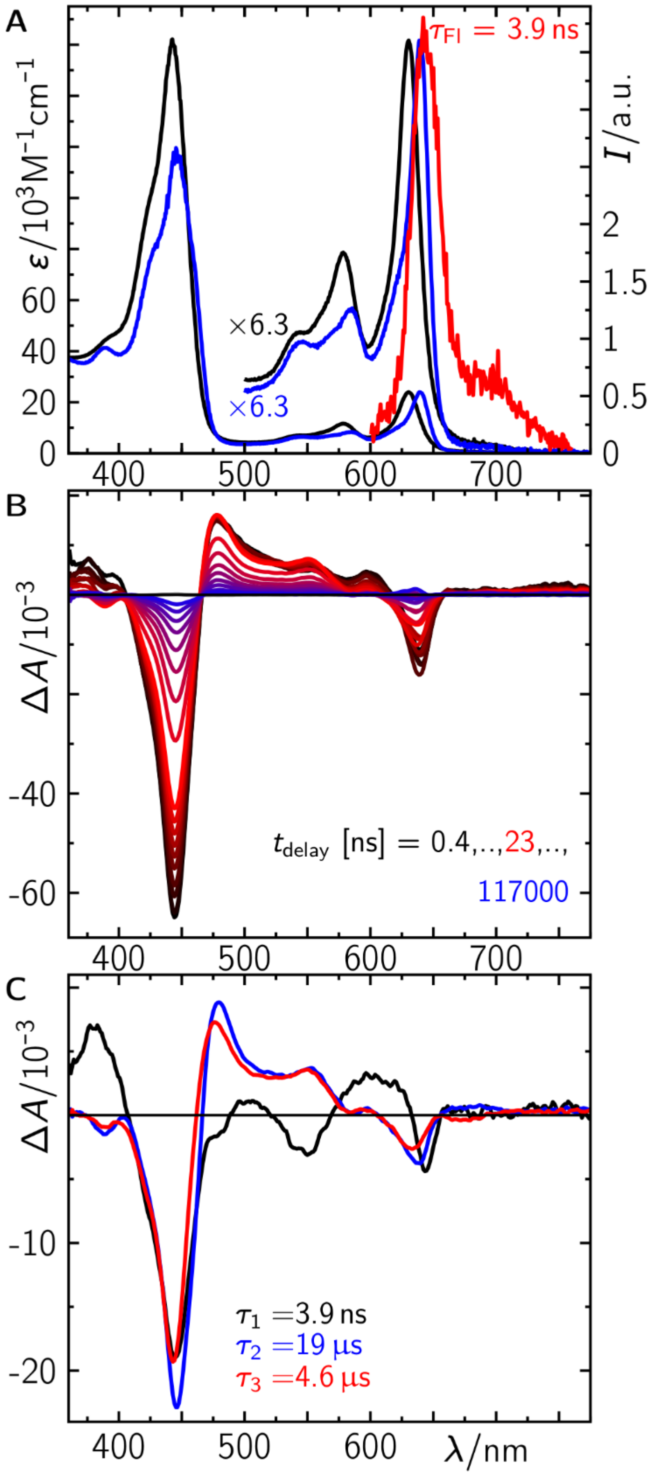

Figure S9. Spectrally and temporally-resolved emission spectra of POR-bound PChlide in the pseudo-ternary complex (PChlide/POR/NADP$^+$) in buffer excited at 450 nm. The left-hand false colour plot represents the raw data. The right-hand time trace represents the summed data (black circles), the corresponding global fit (red lines) and the resulting residuals (weighted by the square-root of the counted events at each time-point accounting for Poissonian statistics) in the spectral range as indicated by the dashed black rectangle on top of the false colour plot. The lifetime is 3.9 ns. The grey dashed rectangle indicates a spectral region, where artificial signals were counted due to overmodulation on the data image caused by the intense laser. This was replaced by the fit during the global fitting procedure as described elsewhere.[8]

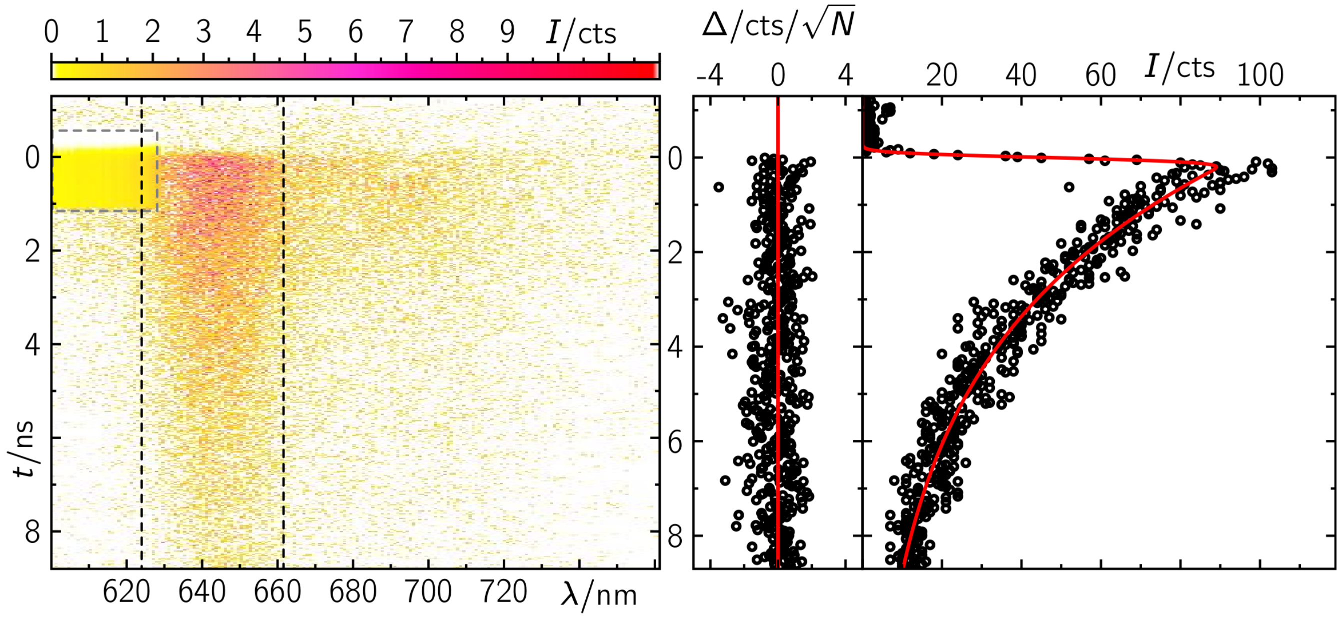

Figure S10. Stationary absorption (black and blue) spectra of POR-bound PChlide in the ternary complexes PChlide/POR/NADPH (**A**) and PChlide/POR-C226S/NADPH (**B**) in buffer. For comparison, the stationary absorption spectrum of unbound PChlide in buffer is shown (grey and light-blue).

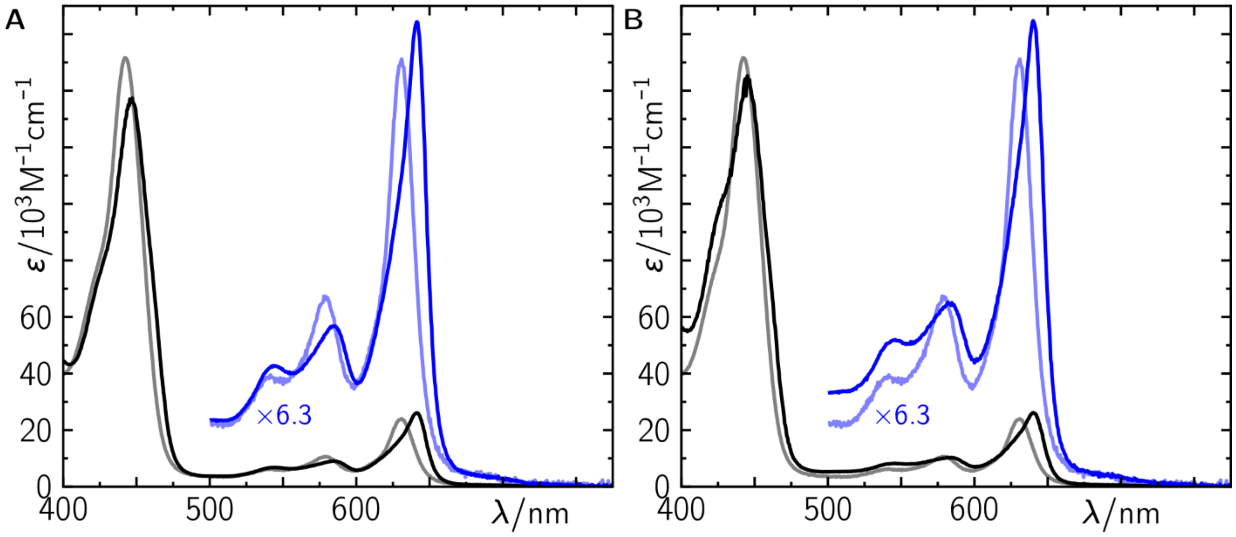

Figure S11. Time-resolved difference absorption spectra of POR-bound PChlide in the ternary complexes PChlide/POR/NADPH (**A**, **B1-2**) and PChlide/POR-C226S/NADPH (**C**, **D1-2**) in buffer recorded in the first 50 ps after photo-excitation into the Q-band (λ_{exc} = 640 nm, $^1P_1 \leftarrow {}^1P_0$ transition). **A** and **C**: Selected TA spectra at time delays up to 50 ps. **B1-2**: Changes of the band area and width of the excited state absorption signal at *ca.* 470 nm (black circles), respectively, obtained from fitting Gaussians to each difference absorption spectrum at different delay times (an exemplary fit at *t* = 1 ps is shown in the inset of panel **A**). **D1-2**: Changes of the band position and width, respectively, of the excited state absorption signal at *ca.* 475 nm (black circles) obtained from fitting Gaussians to each

difference absorption spectrum (an exemplary fit at t = 1 ps is shown in the inset of panel **C**). The red lines in panels **B1-2** and **D1-2** represent global fits to all obtained parameters of the difference spectra describing Gaussians (for further details see experimental section).

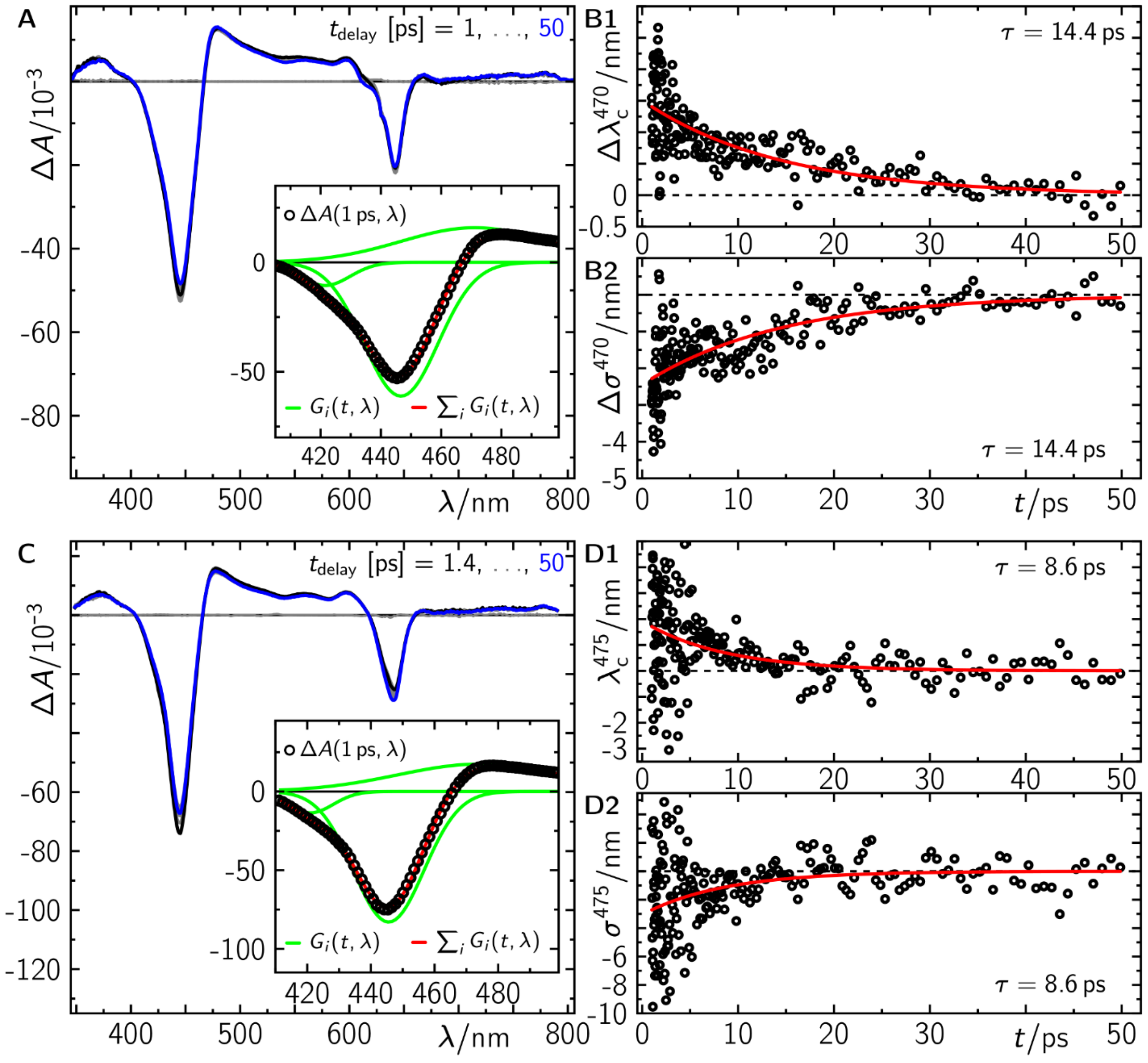

Figure S12. Spectrally and temporally-resolved difference absorption spectra of the ternary complexes PChlide/NADPD/POR (**A-B**), PChlide/NADPD/POR-C226S (**C-D**) in buffer, and PChlide/NADPH/POR-C226S in deuterated buffer (**E-F**). The left-hand false colour plot represents the raw data. The right-hand spectra represent the corresponding global fits. The grey dashed rectangle indicates spectral regions due to laser scatter. This was replaced by the fit during the global fitting procedure as described elsewhere.[8] Samples shown in **A** and **E** were excited at 640 nm, and the sample shown in **C** was excited at 450 nm.

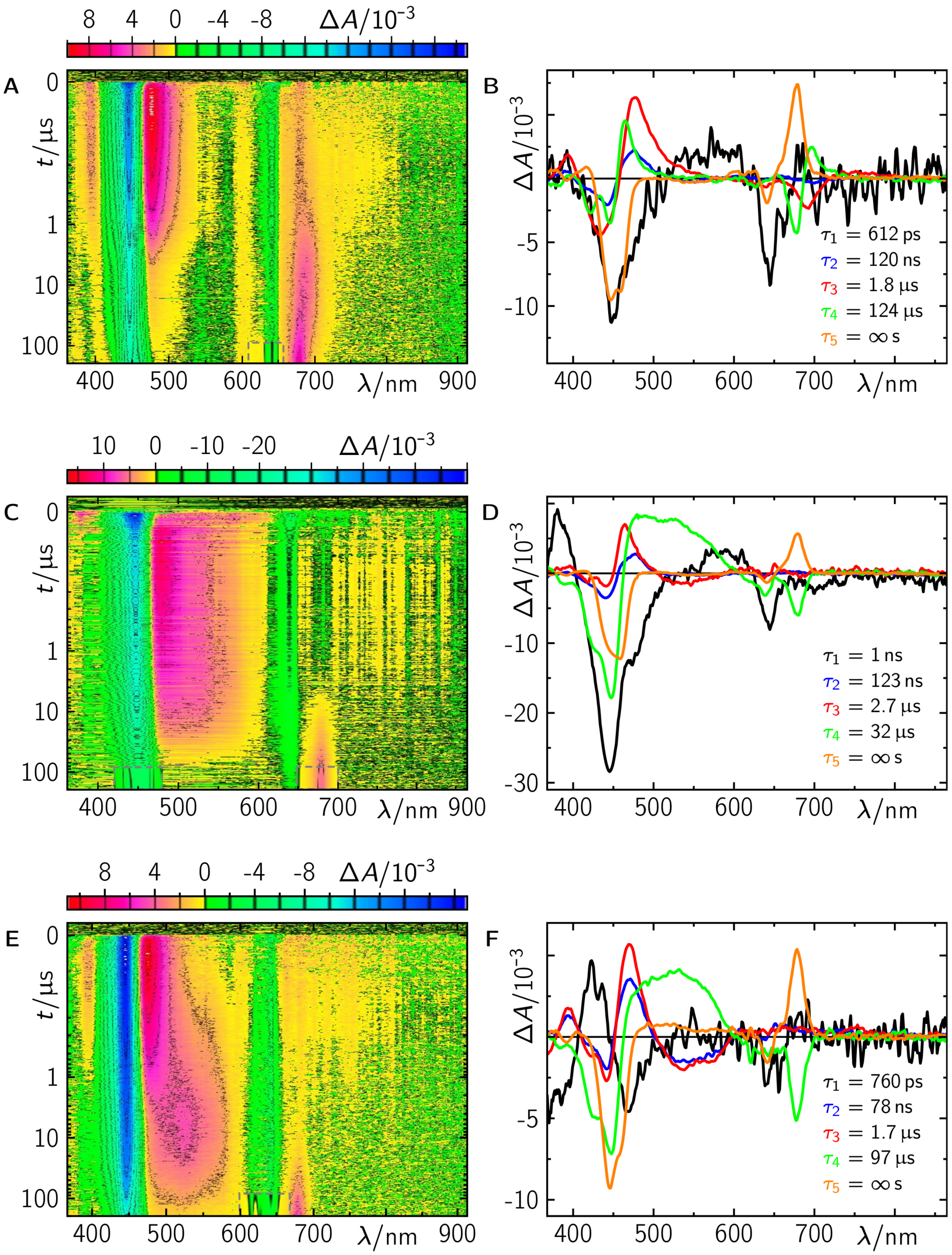

Figure S13. SAS of PChlide in buffer in the $^1\mathrm{P}_1$ state, $^1\mathrm{P}_n \leftarrow {}^1\mathrm{P}_1$ transitions, (**A**), and in the $^3\mathrm{P}_1$ state, $^3\mathrm{P}_n \leftarrow {}^3\mathrm{P}_1$ transitions, (**B**). The contribution of the ground state spectrum, c_0, was varied between 0.03 (red) and 0.12 (green). The preferred value of c_0 is 0.09. The triplet yield, $\Phi_{^3\mathrm{P}_1}$, was varied between 0.3 (red) and 0.8 (green). The preferred value of $\Phi_{^3\mathrm{P}_1}$ is 0.55.

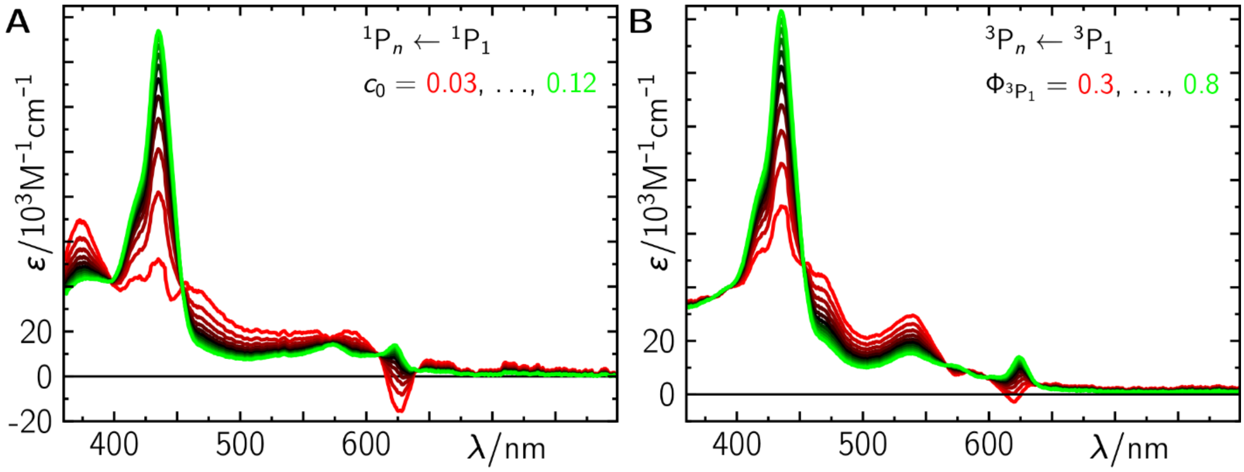

Figure S14. SAS of PChlide bound to the pseudo-ternary complex (PChlide/POR/NADP$^+$) in the $^1P_1$ state, $^1P_n \leftarrow {}^1P_1$ transitions, (**A**), and in the $^3P_1$ state, $^3P_n \leftarrow {}^3P_1$ transitions, (**B**). The contribution of the ground state spectrum, c_0, was varied between 0.02 (red) and 0.04 (green). The preferred value of c_0 is 0.03. The triplet yield, $\Phi_{^3P_1}$, was varied between 0.3 (red) and 0.8 (green). The preferred value of $\Phi_{^3P_1}$ is 0.55.

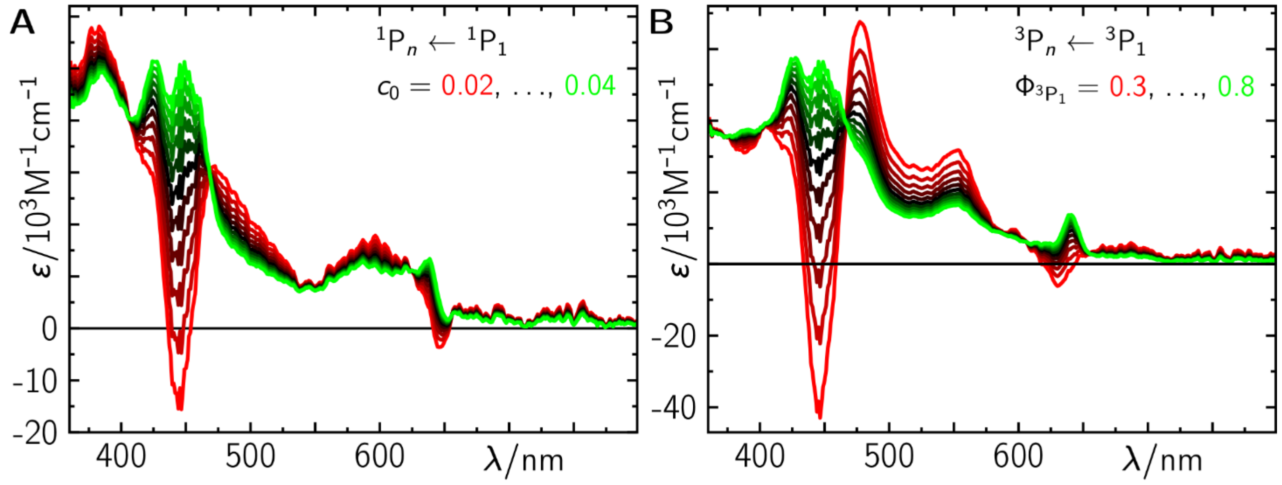

Figure S15. SAS of PChlide bound to the ternary complex (PChlide/POR/NADPH) in the $^1\mathrm{P}_1$ state, $^1\mathrm{P}_n \leftarrow {}^1\mathrm{P}_1$ transitions, (**A**), the first intermediate $^2\mathrm{I}_0^{(1)}$ state, $^2\mathrm{I}_n^{(1)} \leftarrow {}^2\mathrm{I}_0^{(1)}$ transitions, (**B**), the second intermediate $^2\mathrm{I}_0^{(2)}$ state, $^2\mathrm{I}_n^{(2)} \leftarrow {}^2\mathrm{I}_0^{(2)}$ transitions, (**C**), the third intermediate $^1\mathrm{I}_0^{(3)}$ state, $^1\mathrm{I}_n^{(3)} \leftarrow {}^1\mathrm{I}_0^{(3)}$ transitions, (**D**), and the final product Chlide $^1\mathrm{C}_0$ state, $^1\mathrm{C}_n \leftarrow {}^1\mathrm{C}_0$ transitions, (**E**). The contribution of the ground state spectrum, c_0, was varied between 0.04 (red) and 0.095 (green). The preferred value of c_0 is 0.067. The yield for the first intermediate, $\Phi_{^2\mathrm{I}_0^{(1)}}$, was varied between 0.7 (red) and 0.97 (green). The preferred value of $\Phi_{^2\mathrm{I}_0^{(1)}}$ is 0.83. The yield for the second intermediate, $\Phi_{^2\mathrm{I}_0^{(2)}}$, was varied between 0.4 (red) and 0.76 (green). The preferred value of $\Phi_{^2\mathrm{I}_0^{(2)}}$ is 0.5. The yield for the third

intermediate, $\Phi_{^2\mathrm{I}_0^{(3)}}$, was varied between 0.5 (red) and 0.95 (green). The preferred value of $\Phi_{^2\mathrm{I}_0^{(3)}}$ is 1.0. The yield for the final product, $\Phi_{^1\mathrm{C}_0}$, was varied between 0.8 (red) and 0.98 (green). The preferred value of $\Phi_{^1\mathrm{C}_0}$ is 1.0. Thus, the total quantum yield of Chlide formation is $\Phi_{^1\mathrm{C}_0}^{\mathrm{total}} = \Phi_{^2\mathrm{I}_0^{(1)}}\Phi_{^2\mathrm{I}_0^{(2)}}\Phi_{^1\mathrm{I}_0^{(3)}}\Phi_{^1\mathrm{C}_0} = 0.43$. Panel **F** shows the SAS for the preferred values.

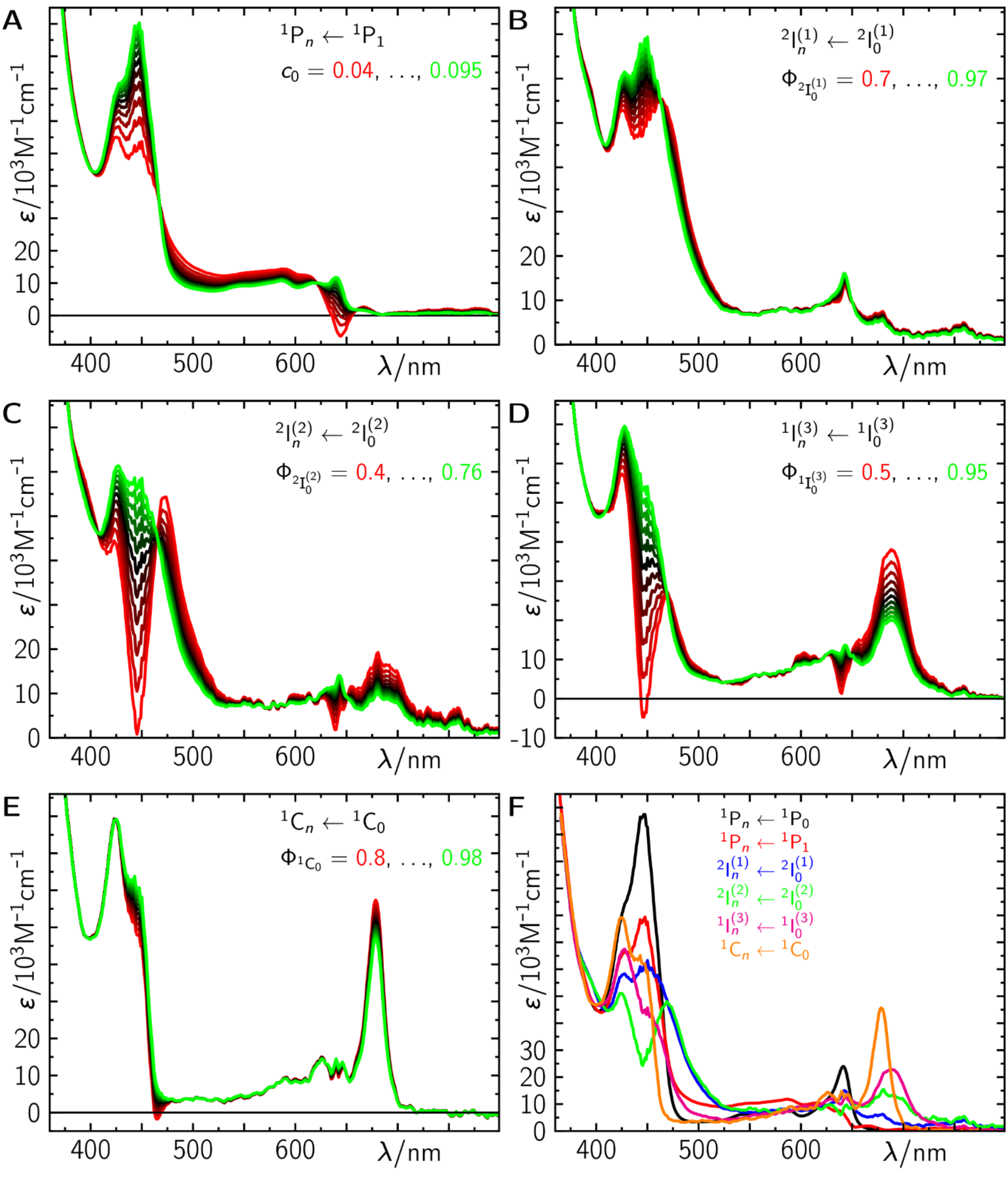

Figure S16. SAS of PChlide bound to the ternary complex (PChlide/POR-C226S/NADPH) in the $^1\mathrm{P}_1$ state, $^1\mathrm{P}_n \leftarrow {}^1\mathrm{P}_1$ transitions, (**A**), the first intermediate $^2\mathrm{I}_0^{(1')}$ state, $^2\mathrm{I}_n^{(1')} \leftarrow {}^2\mathrm{I}_0^{(1')}$ transitions, (**B**), the second intermediate $^2\mathrm{I}_0^{(2')}$ state, $^2\mathrm{I}_n^{(2')} \leftarrow {}^2\mathrm{I}_0^{(2')}$ transitions, (**C**), the third intermediate $^1\mathrm{I}_0^{(3')}$ state, $^1\mathrm{I}_n^{(3')} \leftarrow {}^1\mathrm{I}_0^{(3')}$ transitions, (**D**), and the final product Chlide $^1\mathrm{C}_0$ state, $^1\mathrm{C}_n \leftarrow {}^1\mathrm{C}_0$ transitions, (**E**). The contribution of the ground state spectrum, c_0, was varied between 0.07 (red) and 0.25 (green). The preferred value of c_0 is 0.11. The yield for the first intermediate, $\Phi_{^2\mathrm{I}_0^{(1')}}$, was varied between 0.66 (red) and 0.97 (green). The preferred value of $\Phi_{^2\mathrm{I}_0^{(1')}}$ is 0.83. The yield for the second intermediate, $\Phi_{^2\mathrm{I}_0^{(2')}}$, was varied between 0.45 (red) and 0.95 (green). The preferred value of $\Phi_{^2\mathrm{I}_0^{(2')}}$ is 0.7. The

yield for the third intermediate, $\Phi_{^2I_0^{(3')}}$, was varied between 0.6 (red) and 0.96 (green). The preferred value of $\Phi_{^2I_0^{(3')}}$ is 0.75. The yield for the final product, $\Phi_{^1C_0}$, was varied between 0.65 (red) and 0.97 (green). The preferred value of $\Phi_{^1C_0}$ is 1.0. Thus, the total quantum yield of Chlide formation is $\Phi^{\mathrm{total}}_{^1C_0} = \Phi_{^2I_0^{(1')}}\Phi_{^2I_0^{(2')}}\Phi_{^1I_0^{(3')}}\Phi_{^1C_0} = 0.44$. Panel **F** shows the SAS for the preferred values.

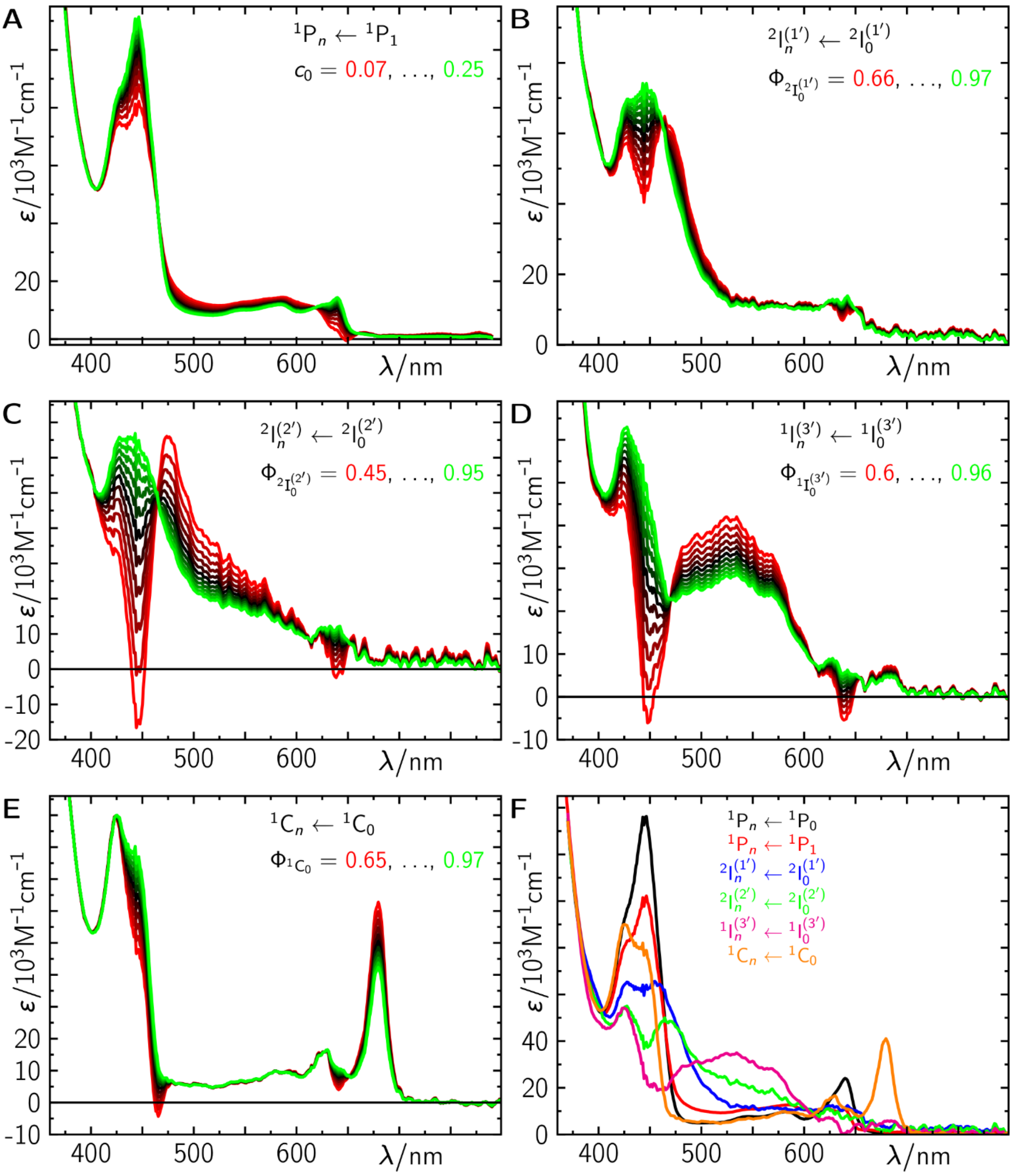

Figure S17. Calculated SAS of four possible stereoconformers of the PChlide anion in the gas phase (**A**-**B**), in water (**C**-**D**), and in cyclohexane (**E**-**F**). In the structures of the corresponding PChlide anion stereoconformer the position of the hydride ion is highlighted in magenta (black spectra when attached to C_{17}; red spectra when attached to C_{18}). $^1I_n^{(3)} \leftarrow {}^1I_0^{(3)}$ black lines in **A**, **C**, and **E**. $^1I_n^{(3')} \leftarrow {}^1I_0^{(3')}$ red lines in **A**, **C**, and **E**. $^1I_n^{(3'')} \leftarrow {}^1I_0^{(3'')}$ black lines in **B**, **D**, and **F**. $^1I_n^{(3''')} \leftarrow {}^1I_0^{(3''')}$ red lines in **B**, **D**, and **F**. A Doppler broadening profile with a width of 20 nm was applied on top of each transition.

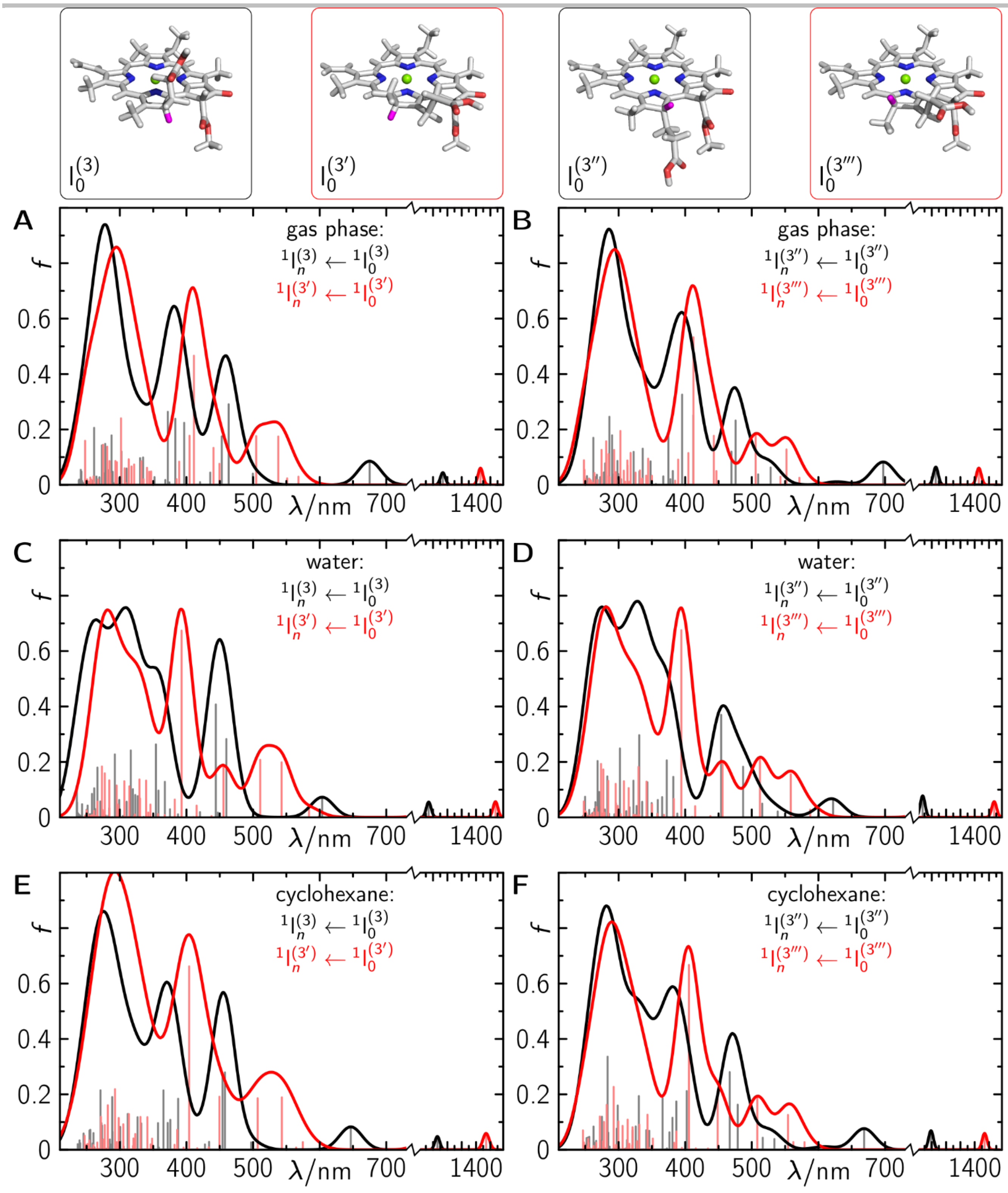

Figure S18. Attempt to visualize the binding situation in POR in order to explain the spectroscopic results. Models of the POR/NADPH/PChlide (**A**) and POR-C226S/NADPH/PChlide (**B**) complexes and their corresponding Root-Mean-Square-Deviation (RMSD) of the protein atoms along the 50 ns classical molecular mechanics simulation trajectories (**C**). **D**: Circular dichroism (CD) absorption spectra of Chlide formed either by POR wild type (black line) or by the POR-C226S variant (red line).

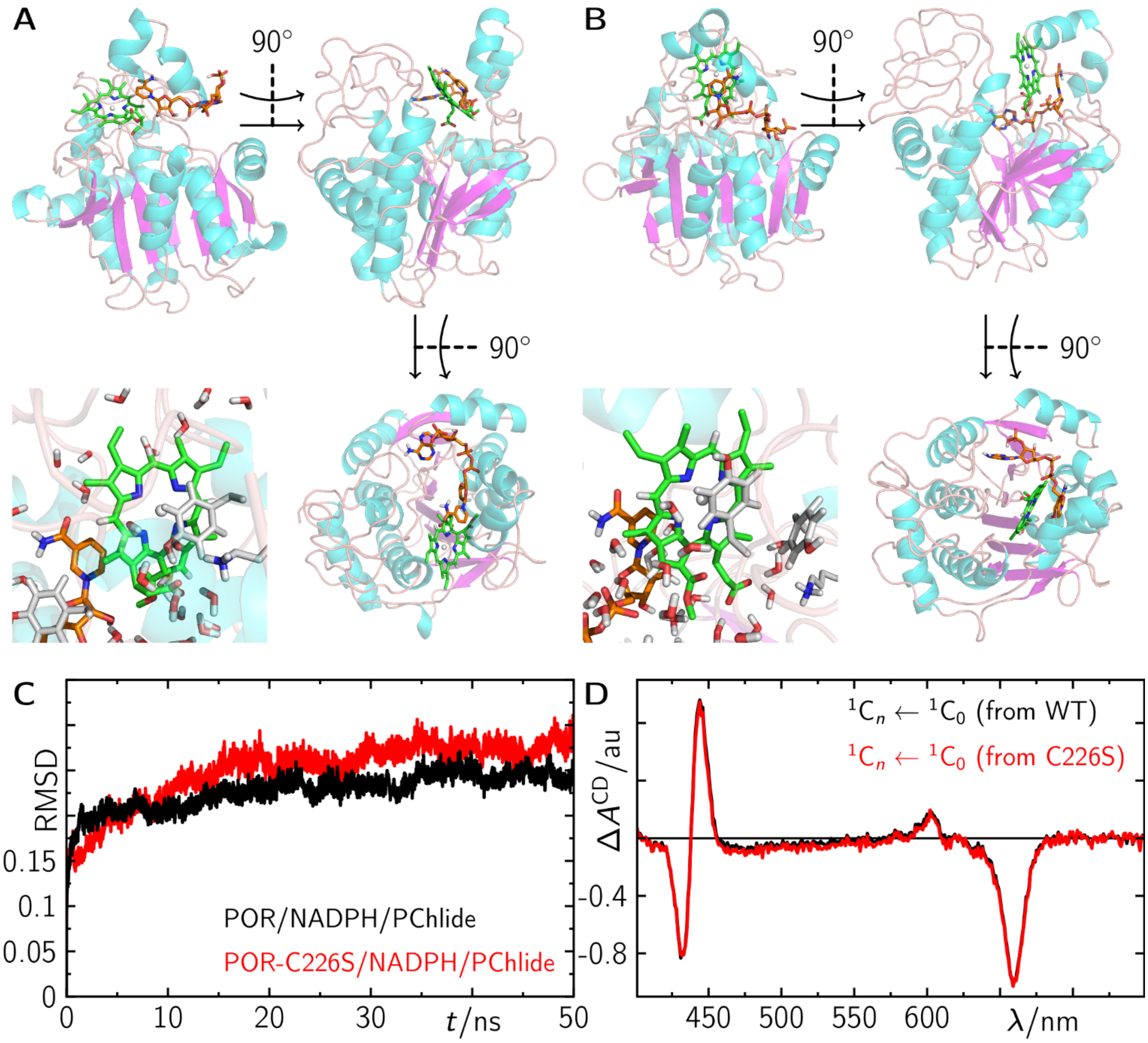

Figure S19. Quantum chemical calculation of PChlide in the gas phase. The PChlide geometry was optimized in its singlet ground state and the vertical excitation energies into the first excited singlet state as well as into two lowest triplet states were calculated *via* the TD-DFT method. The molecular orbitals (MOs) involved in the corresponding transitions into the excited states of the R-B3LYP reference for the singlet state calculations are shown on the left and those of the R-BVWN5 reference for the triplet state are shown on the right. In the middle, the electron densities of the involved MOs are shown. Black surfaces represent the initial density and magenta surfaces represent the final density. Green arrows indicate the dipole moments of the corresponding states and red arrows indicate the transition dipole moments of the corresponding transition.

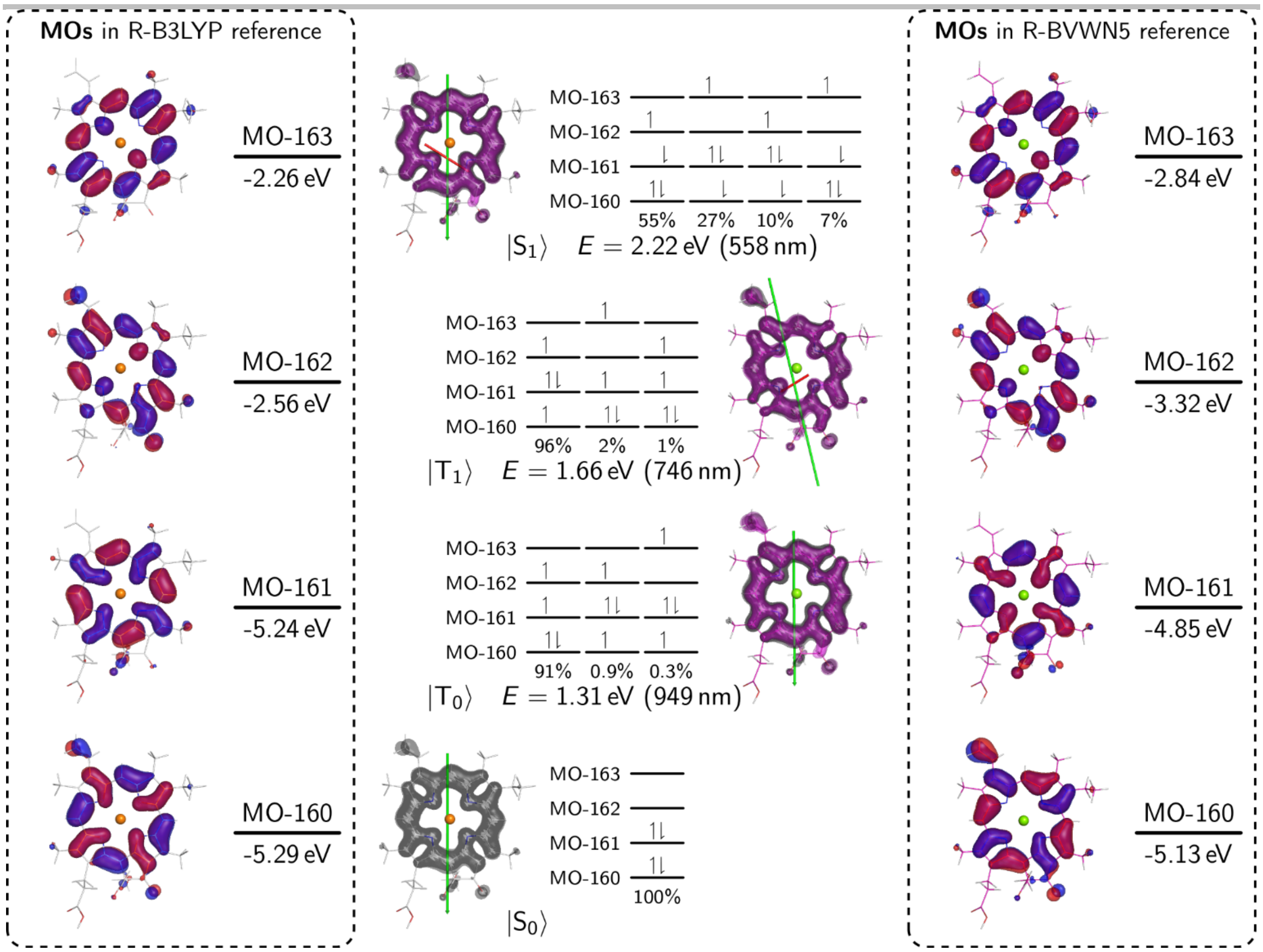

Figure S20. Quantum chemical calculations of PChlide comparing electron densities of ground and first excited state in the gas phase, cyclohexane, and water. The PChlide geometry was optimized in its first excited singlet state *via* the TD-DFT method. The electron densities of the involved MOs are shown. Black surfaces represent the initial density and magenta surfaces represent the final density.

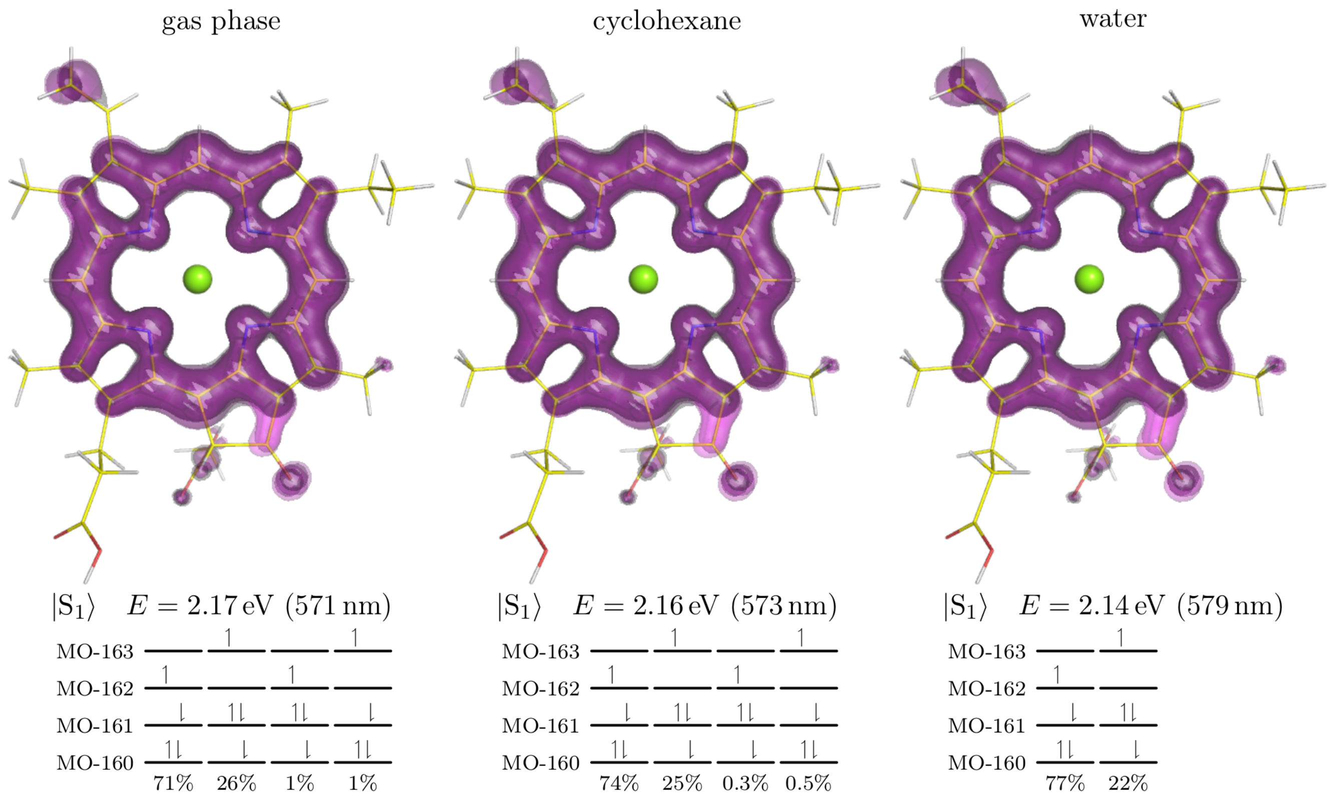

Figure S21. Residual plot for global exponential fit on data obtained for the ternary complex PChlide/-NADPH/POR. The residuals obtained for all other data sets are of comparable quality. The left-hand false colour plot represents the entire residual data matrix. The right-hand temporal slices correspond to the indicated colour coded dashed lines on top of the false colour plot. The black rectangle indicates spectral regions due to laser scatter. This was replaced by the fit during the global fitting procedure as described elsewhere.[8]

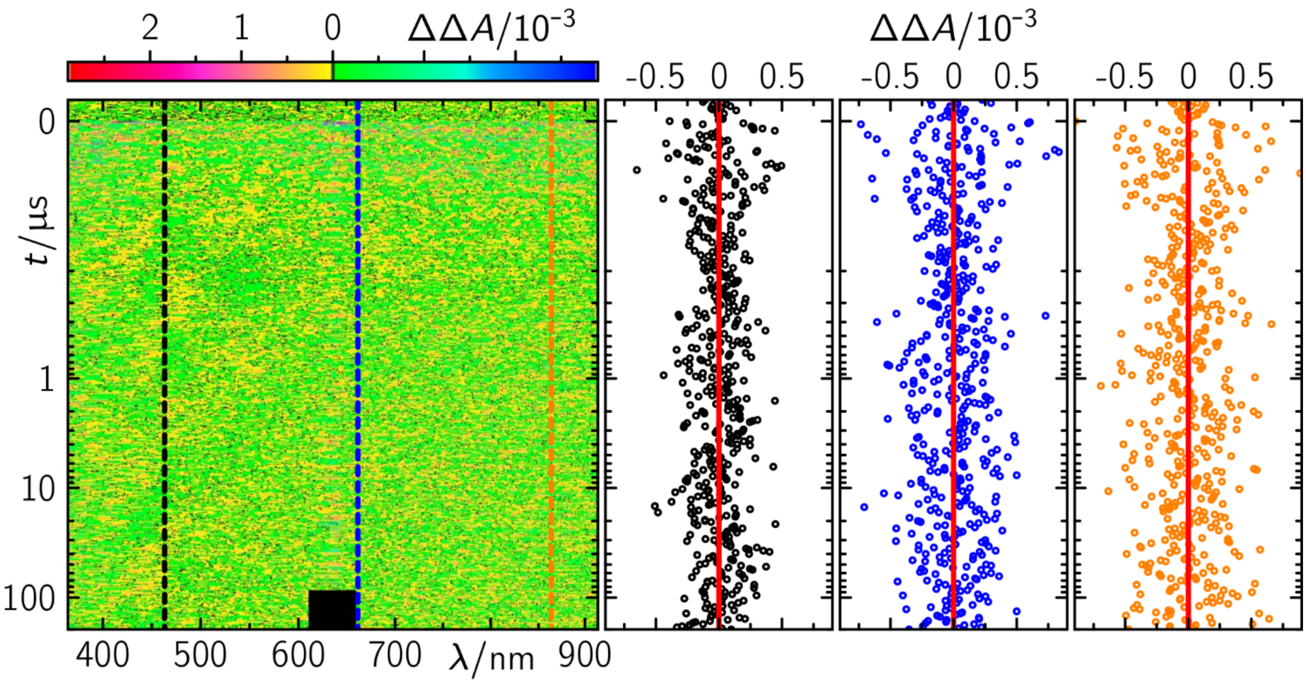

References

[1] D. J. Heyes, A. V. Ruban, H. M. Wilks, C. N. Hunter, *Proceedings of the National Academy of Sciences* **2002**, *99*, 11145–11150.
[2] C. R. Pudney, S. Hay, N. S. Scrutton, in *Flavins Flavoproteins: Methods Protocols* (Eds.: S. Weber, E. Schleicher), Springer New York, New York, NY, **2014**, pp. 161–175.
[3] H. Klement, M. Helfrich, U. Oster, S. Schoch, W. Rüdiger, *European Journal of Biochemistry* **1999**, *265*, 862–874.
[4] C. E. Cameron, S. J. Benkovic, *Biochemistry* **1997**, *36*, 15792–15800.
[5] A. Garrone, N. Archipowa, P. F. Zipfel, G. Hermann, B. Dietzek, *Journal of Biological Chemistry* **2015**, *290*, 28530–28539.
[6] B. R. K. Menon, P. A. Davison, C. N. Hunter, N. S. Scrutton, D. J. Heyes, *Journal of Biological Chemistry* **2010**, *285*, 2113–2119.
[7] R. J. Kutta, U. Kensy, B. Dick, in *Chemical Photocatalysis* (Ed.: B. König), De Gruyter, **2013**, pp. 295–318.
[8] R. J. Kutta, T. Langenbacher, U. Kensy, B. Dick, *Applied Physics B* **2013**, *111*, 203–216.
[9] K. Lanzl, M. v. Sanden-Flohe, R. J. Kutta, B. Dick, *Physical Chemistry Chemical Physics* **2010**, *12*, 6594–6604.
[10] K. Arnold, L. Bordoli, J. Kopp, T. Schwede, *Bioinformatics* **2006**, *22*, 195–201.
[11] L. Baugh, I. Phan, D. W. Begley, M. C. Clifton, B. Armour, D. M. Dranow, B. M. Taylor, M. M. Muruthi, J. Abendroth, J. W. Fairman, et al., *Tuberculosis* **2015**, *95*, 142–148.
[12] D. J. Miller, Y.-M. Zhang, C. O. Rock, S. W. White, *Journal of Biological Chemistry* **2006**, *281*, 18025–18032.
[13] A. Sali, T. L. Blundell, *Journal of Molecular Biology* **1993**, *234*, 779–815.
[14] B. Hess, C. Kutzner, D. van der Spoel, E. Lindahl, *Journal of Chemical Theory and Computation* **2008**, *4*, 435–447.
[15] A. A. Granovsky, *Firefly version 8, www http://classic.chem.msu.su/gran/firefly/index.html* **1997**.
[16] M. W. Schmidt, K. K. Baldridge, J. A. Boatz, S. T. Elbert, M. S. Gordon, J. H. Jensen, S. Koseki, N. Matsunaga, K. A. Nguyen, S. Su, et al., *Journal of Computational Chemistry* **1993**, *14*, 1347–1363.
[17] M. J. McFarlane, C. N. Hunter, D. J. Heyes, *Photochem. Photobiol. Sci.* **2005**, *4*, 1055–1059.
[18] G.-J. Zhao, K.-L. Han, *Biophysical Journal* **2008**, *94*, 38–46.
[19] B. Dietzek, S. Tschierlei, G. Hermann, A. Yartsev, T. Pascher, V. Sundström, M. Schmitt, J. Popp, *ChemPhysChem* **2009**, *10*, 144–150.
[20] O. A. Sytina, I. H. M. van Stokkum, D. J. Heyes, C. N. Hunter, R. van Grondelle, M. L. Groot, *The Journal of Physical Chemistry B* **2010**, *114*, 4335–4344.
[21] N. S. Scrutton, M. Louise Groot, D. J. Heyes, *Phys. Chem. Chem. Phys.* **2012**, *14*, 8818–8824.
[22] O. A. Sytina, I. H. M. van Stokkum, D. J. Heyes, C. N. Hunter, M. L. Groot, *Phys. Chem. Chem. Phys.* **2012**, *14*, 616–625.
[23] G. Brandariz-de-Pedro, D. J. Heyes, S. J. O. Hardman, M. Shanmugam, A. R. Jones, S. Weber, D. Nohr, N. S. Scrutton, A. J. Fielding, *The Journal of Physical Chemistry Letters* **2017**, *8*, 1219–1223.
[24] D. J. Heyes, S. J. O. Hardman, T. M. Hedison, R. Hoeven, G. M. Greetham, M. Towrie, N. S. Scrutton, *Angewandte Chemie International Edition* **2015**, *54*, 1512–1515.
[25] D. J. Heyes, S. J. O. Hardman, D. Mansell, A. N'i Cheallaigh, J. M. Gardiner, L. O. Johannissen, G. M. Greetham, M. Towrie, N. S. Scrutton, *The Journal of Physical Chemistry B* **2017**, *121*, 1312–1320.
[26] B. Dietzek, W. Kiefer, G. Hermann, J. Popp, M. Schmitt, *The Journal of Physical Chemistry B* **2006**, *110*, 4399–4406.
[27] B. Dietzek, W. Kiefer, A. Yartsev, V. Sundström, P. Schellenberg, P. Grigaravicius, G. Hermann, J. Popp, M. Schmitt, *ChemPhysChem* **2006**, *7*, 1727–1733.
[28] B. Dietzek, R. Maksimenka, T. Siebert, E. Birckner, W. Kiefer, J. Popp, G. Hermann, M. Schmitt, *Chemical Physics Letters* **2004**, *397*, 110–115.
[29] B. Dietzek, S. Tschierlei, R. Hanf, S. Seidel, A. Yartsev, M. Schmitt, G. Hermann, J. Popp, *Chemical Physics Letters* **2010**, *492*, 157–163.
[30] M. Schmitt, B. Dietzek, G. Hermann, J. Popp, *Laser & Photonics Reviews* **2007**, *1*, 57–78.
[31] B. Mysliwa-Kurdziel, J. Kruk, K. Strzalka, *Photochemistry and Photobiology* **2004**, *79*, 62–67.
[32] C. Franco, J. I. Olmsted, *Talanta* **1990**, *37*, 905–909.
[33] M. A. El-Sayed, *Accounts of Chemical Research* **1968**, *1*, 8–16.
[34] N. J. Turro, V. Ramamurthy, J. C. Scaiano, *Modern Molecular Photochemistry Organic Molecules*, University Science Books, **2010**.
[35] H. A. C. Montgomery, N. S. Thom, A. Cockburn, *Journal of Applied Chemistry* **1964**, *14*, 280–296.

[36] J. Nomata, T. Kondo, T. Mizoguchi, H. Tamiaki, S. Itoh, Y. Fujita, *Scientific Reports* **2014**, *4*, 5455–.
[37] D. J. Heyes, M. Sakuma, S. P. de Visser, N. S. Scrutton, *Journal of Biological Chemistry* **2009**, *284*, 3762–3767.

Author Contributions

All raw data from experiments are available from the authors on request. N.A. and R.J.K. contributed equally to the work. N.A. and R.J.K. wrote the paper with input from all of the authors. All samples were prepared by N.A.. R.J.K. wrote the programs for data analysis and performed the molecular simulations and quantum chemical calculations. N.A. and R.J.K. designed the experiments. All experimental work, data analysis, as well as, data interpretation was conducted by N.A. and R.J.K.. Results were discussed by all authors. The project was conceived and funding acquired by D.J.H. and N.S.S.
